# Metformin Resensitizes Sorafenib-Resistant HCC Cells Through AMPK-Dependent Autophagy Activation

**DOI:** 10.3389/fcell.2020.596655

**Published:** 2021-01-21

**Authors:** Hong-Yue Lai, Hsin-Hwa Tsai, Chia-Jui Yen, Liang-Yi Hung, Ching-Chieh Yang, Chung-Han Ho, Hsin-Yin Liang, Feng-Wei Chen, Chien-Feng Li, Ju-Ming Wang

**Affiliations:** ^1^Department of Biotechnology and Bioindustry Sciences, College of Bioscience and Biotechnology, National Cheng Kung University, Tainan, Taiwan; ^2^Department of Medical Research, Chi Mei Medical Center, Tainan, Taiwan; ^3^Department of Pathology, Chi Mei Medical Center, Tainan, Taiwan; ^4^Department of Oncology, National Cheng Kung University Hospital, Tainan, Taiwan; ^5^College of Medicine, National Cheng Kung University, Taipei, Taiwan; ^6^College of Medicine, Graduate Institute of Medicine, Kaohsiung Medical University, Kaohsiung, Taiwan; ^7^Department of Radiation Oncology, Chi-Mei Medical Center, Tainan, Taiwan; ^8^Department of Pharmacy, Chia-Nan University of Pharmacy and Science, Tainan, Taiwan; ^9^Department of Hospital and Health Care Administration, Chia Nan University of Pharmacy and Science, Tainan, Taiwan; ^10^International Center for Wound Repair and Regeneration, National Cheng Kung University, Tainan, Taiwan; ^11^Institute of Basic Medical Sciences, College of Medicine, National Cheng Kung University, Tainan, Taiwan; ^12^National Institute of Cancer Research, National Health Research Institute, Tainan, Taiwan; ^13^Institute of Clinical Medicine, Kaohsiung Medical University, Kaohsiung, Taiwan; ^14^Department of Biotechnology, Southern Taiwan University of Science and Technology, Tainan, Taiwan; ^15^College of Medical Science and Technology, Graduate Institute of Medical Sciences, Taipei Medical University, Taipei, Taiwan

**Keywords:** sorafenib, metformin, autophagy, AMPK, CEBPD

## Abstract

Despite the activation of autophagy may enable residual cancer cells to survive and allow tumor relapse, excessive activation of autophagy may eventually lead to cell death. However, the details of the association of autophagy with primary resistance in hepatocellular carcinoma (HCC) remain less clear. In this study, cohort analysis revealed that HCC patients receiving sorafenib with HBV had higher mortality risk. We found that high epidermal growth factor receptor (EGFR) expression and activity may be linked to HBV-induced sorafenib resistance. We further found that the resistance of EGFR-overexpressed liver cancer cells to sorafenib is associated with low activity of AMP-activated protein kinase (AMPK) and CCAAT/enhancer binding protein delta (CEBPD) as well as insufficient autophagic activation. In response to metformin, the AMPK/cAMP-response element binding protein (CREB) pathway contributes to CEBPD activation, which promotes autophagic cell death. Moreover, treatment with metformin can increase sorafenib sensitivity through AMPK activation in EGFR-overexpressed liver cancer cells. This study suggests that AMPK/CEBPD-activated autophagy could be a potent strategy for improving the efficacy of sorafenib in HCC patients.

## Introduction

Sorafenib is a multi-kinase inhibitor that mainly targets Raf kinases and receptor tyrosine kinases, including vascular endothelial growth factor receptor (VEGFR)-2/3, platelet-derived growth factor receptor (PDGFR)-β, FMS-like tyrosine kinase 3 (Flt3), and c-Kit (CD117) (Cervello et al., 2012), which are involved in tumor angiogenesis and progression. However, the overall outcomes for patients with advanced hepatocellular carcinoma (HCC) are discouraging and distinct tumor cells exhibit diverse degrees of sensitivity to sorafenib. Therefore, a precise understanding of the mechanism of resistance is critical to develop personalized medicine strategies for HCC patients.

Autophagy is a highly conserved intracellular degradation process that can be enhanced when cancer cells face environmental stresses such as nutritional deficiency and even chemotherapy. Autophagy induced by hepatitis B virus (HBV)/hepatitis C virus (HCV) has been suggested to support viral replication and contributes to HCC progression (Wang et al., 2014; Wu et al., 2014; Khan et al., 2018). Epidermal growth factor receptor (EGFR) is overexpressed and activated in more than half of HCC patients (Buckley et al., 2008). A combination of EGFR inhibitor and sorafenib was assessed as a rational therapeutic strategy for HCC (Zhu et al., 2017), but the preclinical results were far from satisfactory. Some studies showed that autophagy induced by EGFR inhibitors is cytoprotective, and the combination of EGFR inhibitors with autophagy inhibitors might be beneficial (Wang et al., 2019). Despite autophagy is involved in a survival mechanism, excessive activation of autophagy could eventually lead to cell death (Liu and Levine, 2015). Autophagy is also responsive to sorafenib stress and strengthens the sorafenib-induced death of cancer cells (Park et al., 2010). Therefore, the complex role of autophagy should be clarified, which may be important to precisely regulate the levels of autophagy to control HCC.

CCAAT/enhancer binding protein delta (CEBPD) is a transcription factor that responds to various external stimuli, including the proinflammatory cytokines IL-1β and TNFα (Chang et al., 2012), stress (O'Rourke et al., 1997), growth factors (Wang et al., 2005), and anti-cancer chemotherapy drugs (Li et al., 2015; Chu et al., 2017; Tsai et al., 2017). CEBPD is thought to be a potent tumor suppressor, and its expression is downregulated in several cancers, including breast cancer (Sivko and DeWille, 2004), leukemia (Agrawal et al., 2007), cervical cancer (Pan et al., 2010), and hepatocellular carcinoma. We previously demonstrated that epigenetic regulation contributes to CEBPD inactivation in cancers (Ko et al., 2008) and that strong CEBPD activation can strengthen the death of cancer cells via eliminating epigenetic control (Li et al., 2015). However, we also found that inhibition of the EGFR/STAT3/CEBPD axis reverses cisplatin resistance in bladder cancer (Wang et al., 2017). Therefore, the different and sometime paradoxical function of CEBPD appears to be dependent on cell type-specific contexts.

Metformin (dimethylbiguanide) is a current first-line pharmacological treatment for type 2 diabetes. Some studies have further demonstrated that metformin can induce cell arrest and promote cell death (Chen et al., 2013). Metformin can activate autophagy by inhibiting mammalian target of rapamycin (mTOR) directly or indirectly in an AMP-activated protein kinase (AMPK)-dependent manner (Kim and He, 2013; Pernicova and Korbonits, 2014). It has also been suggested that metformin should be applied for therapy for other cancers in addition to HCC, including melanoma and lymphoma, via autophagic activation (Tomic et al., 2011; Shi et al., 2012). However, the molecular details of metformin in overcoming the primary resistance of liver cancer cells to sorafenib remains an open question.

## Materials and Methods

### Clinical Data Analysis

Our nationwide cohort analysis used the Taiwan Cancer Registry (TCR) and National Health Insurance Research Database (NHIRD) to identify diagnosis of HCC and sorafenib prescription (Lu et al., 2017; Chan et al., 2019). The TCR database captures 97% of the cancer cases in Taiwan and also represented a perfect data quality comparing to other well-established cancer registries (Bray and Parkin, 2009; Chiang et al., 2016). To ensure patient privacy, all personal identifying information was removed prior to analysis. This study was approved by the Institutional Review Board of Chi-Mei Medical Center in Taiwan (IRB: 10702-E04).

The International Classification of Diseases, Ninth Revision, Clinical Modification (ICD-9-CM) code 155.0 were used to identify patients diagnosed with HCC between 2012 and 2015 from the TCR database. Information on HBV or HCV infection were obtained for the period from 12 months before until 12 months after HCC diagnosis based on ICD-9-CM diagnosis codes: HBV (070.20, 070.22, 070.30, 070.32) and HCV (070.41, 070.44, 070.51, 070.70, 070.71). Patients with a previous cancer history, a lack of clear demographic and tumor information or aged <18 years were excluded. Finally, a total of 6,628 HCC patients were enrolled in this analysis. For the usage of sorafenib, all patients were reimbursed without co-payment by NHI according to the criteria BCLC advanced stage that were not amenable to either surgical resection or locoregional therapy and Child–Pugh class A liver functional reserve. The prescription of sorafenib is 800 mg (200 mg/tablet) for 2 months. The application needed to be re-evaluated every 2 months for next term of sorafenib usage with imaging evidence showing no disease progression.

The categorical variables were presented as frequency with percentage, and the difference between patients with HBV diagnosed and those without was compared using Pearson's chi-square test. The 1-year mortality risk for HCC patients with different hepatitis B/C virus was estimated using Cox proportional regression analysis adjusted with age, gender, HCC diagnosed to start sorafenib, dosage of sorafenib, comorbidities, and additional therapy after sorafenib such as TACE, RFA, radiation, hepatectomy, and liver transplantations. The stratified analysis was also implemented to investigate the mortality risk among the different duration of sorafenib used. In addition, the estimation of different follow-up period mortality risk was considered. The predicted survival curves were plotted using the results of above Cox regression analysis with adjusted confounding factors. SAS 9.4 for Windows (SAS Institute, Inc., Cary, NC, USA) was used for all statistical analyses. All statistical tests were 2-sided, and *p* < 0.05 was considered statistically significant.

### Cell Culture

The human hepatocellular carcinoma cell lines Huh7 and Hep3B were maintained in Dulbecco's modified Eagle's medium (DMEM) supplemented with 10% fetal bovine serum (FBS), 100 μg/ml streptomycin, and 100 units/ml penicillin at 37°C and 5% CO_2_.

### Lentiviral shRNA Knockdown

The virus was produced from Phoenix Ampho cells using Mirus Bio TransIT-2020 and cotransfected with various short hairpin RNA (shRNA) expression vectors in combination with pMD2.G and psPAX2 vectors and the pLKO.1-shRNA expression vectors. The short interfering RNA sequences targeting LacZ, CEBPD, and AMPK were subcloned into the lentiviral expression vector pLKO.1. The short interfering RNA sequences are as follows: shLacZ (shZ): 5′-CCGGTGTTCGCATTATCCGAACCATCTCGAGATGGTTCGGATAATGCGAACATTTTTG-3′; shCEBPD (shD): 5′-CCGGGCCGACCTCTTCAACAGCAATCTCGAGATTGCTGTTGAAGAGGTCGGCTTTTT-3′; shAMPKα (shKα1): 5′-CCGGTGATTGATGATGAAGCCTTAACTCGAGTTAAGGCTTCATCATCAATCATTTTT-3′; shAMPKα (shKα2): 5′-CCGGCAACTTTACCTGGTTGATAACCTCGAGGTTATCAACCAGGTAAAGTTGTTTT-3′. The expression vectors and shRNAs were obtained from the National RNAi Core Facility located at the Genomic Research Center of Institute of Molecular Biology, Academia Sinica, Taiwan.

### Plasmid Transfection and Reporter Assays

Human CEBPD reporter was constructed in our lab (Wang et al., 2005). The reporter was transfected into Huh7 cells by Turbofect according to the manufacturer's suggestions. Transfectants were cultured in complete medium with or without treatment for 3 h. Luciferase activity was measured in the lysates of transfectants.

### Cell Viability

Huh7 and Hep3B cells were seeded 5^*^10^3^ cells per well in 96-well plates. Cells were treated with various concentrations (0, 2.5, and 5 μM) of sorafenib for 48 h or with the combination of 2.5 μM sorafenib and 5 mM metformin for 48 h. The experimental cells were incubated with diluted MTT reagent [3-(4,5-dimethylthiazol-2-yl)-2,5-diphenyltetrazolium bromide] at 37°C for 3.5 h. The samples were then measured spectrophotometrically at 595 nm by an ELISA plate reader.

### Flow Cytometry Analysis

Huh7 and Hep3B cells were treated with sorafenib for 48 h. Treated and control cells were harvested, washed twice and re-suspended in 500 μl of PBS plus Annexin V-FITC and PI in dark for 15 min at room temperature. The degree of apoptosis was determined as the percentage of cells positive for Annexin V-FITC/PI. For each sample, at least 1 × 10 ^4^ cells were analyzed by FACScan cytometry (CellLab Quanta^TM^ SC, *Beckman Coulter*). The data were determined by three independent experiments.

### Fluorescence Microscopy

The pEGFP-LC3 plasmid was a gift obtained from Dr. Tamotsu Yahsimori and Noboru Mizushima (Kabeya et al., 2000). Huh7 and Hep3B cells transfected with GFP-LC3B plasmid were grown on glass coverslips or treated with sorafenib (2.5 and 5 μM) for 6 h, and then examined under a fluorescence microscope. Images shown are representative of three independent experiments. The fold changes of the average numbers of puncta per positive cells were calculated with 50 individual cells.

### Animal Studies

Male, 6-week-old NOD/SCID mice were obtained from the Laboratory Animal Center of National Cheng Kung University, Tainan, Taiwan. Hep3B cells (5 × 10^6^) in 0.2 ml PBS were inoculated subcutaneously into the right flank of the mice. After 14 days, when macroscopic tumors (50–100 mm^3^) had formed, animals were placed randomly into four groups (*n* = 5 per group) as follows: (1) the control group, which received identical volumes of vehicle; (2) the sorafenib treatment group, which was treated with sorafenib at doses of 15 mg/kg/day; (3) the metformin treatment group, which was treated with 250 mg/kg/day metformin; and (4) the combined treatment group, which was injected with sorafenib combined with metformin. Treatment was given to all groups intraperitoneally every day for 4 weeks. Animal weight and tumor dimensions were measured every 4 days with calipers, and tumor volumes were estimated using two-dimensional measurements of length and width and were calculated with the formula: [*l* × (*w*)^2^] × 0.52, where *l* is length and *w* is width.

### Statistical Analysis

All experiments were repeated at least 3 times, and data were analyzed for statistical significance by two-tailed unpaired Student's *t*-test using Prism 5 software. The data were expressed as the means ± SEM. Differences were considered statistically significant when indicated by asterisks.

## Results

### HBV Is Associated With Sorafenib Resistance in HCC Cells

**Figure 1:**
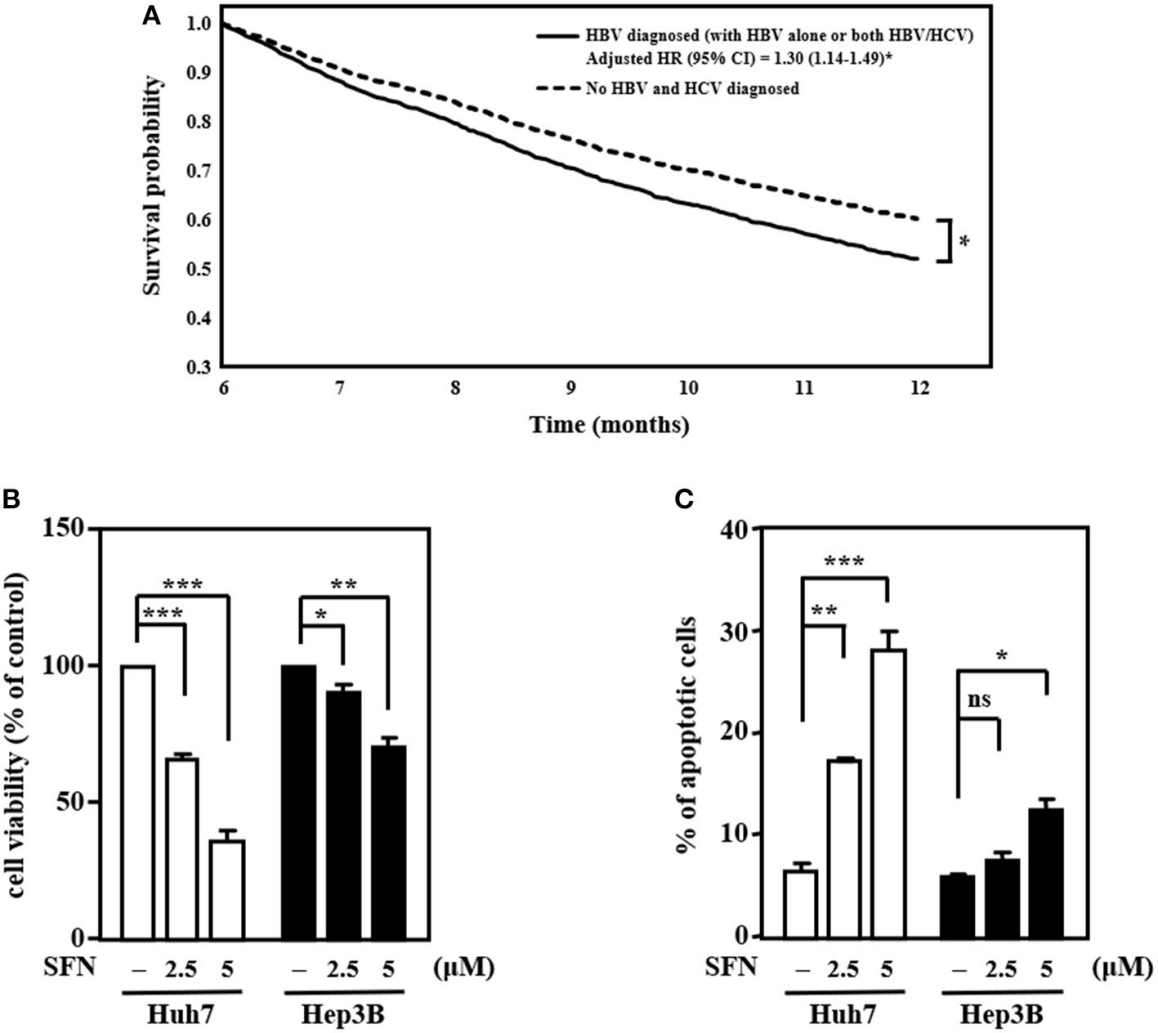
HCC cells with HBV are more resistant than those without HBV to the anti-cancer effects of sorafenib. **(A)** The survival curves for more than 6 months follow-up period. **(B)** Huh7 and Hep3B cells were treated with sorafenib (SFN) at the indicated concentrations for 48 h. The cell viability of the experimental cells was measured by MTT assays after 48 h of sorafenib treatment at the indicated concentrations. **(C)** Huh7 and Hep3B cells were treated with sorafenib at the indicated concentrations for 48 h. Experimental cells were collected after 48 h of sorafenib treatment at the indicated concentrations, stained with Annexin-V/PI, and analyzed by flow cytometry. The data are shown as the mean ± SD. *P < 0.05; **P < 0.01; ***P < 0.001 by Student's *t*-test.

HBV and HCV are major risk factors for HCC and have been associated with therapeutic efficacy. To check the clinical relevance of HBV/HCV with sorafenib resistance in patients with HCC, cohort analysis was performed to identify HCC patients receiving sorafenib with (*n* = 3,389) or without HBV/HCV (*n* = 2,113) (Table 1). After adjusted to potential confounding factors, patients with HBV/HCV increased 10% risk of overall 1-year mortality compared with those without HBV/HCV (Table 2). In addition, patients with HBV/HCV had higher mortality risk at 6–12 months follow-up period than those without HBV/HCV (Table 3), and the estimated survival probability from the hazard function after adjusted to age, gender, HCC diagnosed to start sorafenib, dosage of sorafenib, comorbidities, and additional therapy after sorafenib was plotted as Figure 1A. Interestingly, previous study indicated that sorafenib improved overall survival among patients with HCC who were HCV positive but HBV negative (Jackson et al., 2017), suggesting that HBV might be the major cause of sorafenib resistance. To dissect the presence of HBV in sorafenib resistance, two human HCC cell lines, Huh7 without HBV and Hep3B with an integrated HBV genome, were treated with different concentrations (2.5 and 5 μM) of sorafenib to address this issue. A cell viability assay revealed that Huh7 cells were more sensitive than Hep3B cells to sorafenib (Figure 1B). Furthermore, a cell death assay revealed that sorafenib significantly induced apoptotic cell death in Huh7 cells compared to Hep3B cells (Figure 1C), suggesting that Hep3B cells are intrinsically more resistant than Huh7 cells to sorafenib.

**Table 1:** Clinical information of HCC patients treated with sorafenib with or without HBV/HCV.

**Characteristic**	**HBV diagnosed (with HBV alone or both HBV/HCV)**		**No HBV and HCV diagnosed**		*P*-value^*^
	***N***	**%**	***N***	**%**	
Overall patients	3,389	61.6	2,113	38.4	
**Age groups**					
<35	114	3.36	33	1.56	<0.0001
35–50	769	22.69	195	9.23	
50–65	1,684	49.69	813	38.48	
>65	822	24.25	1,072	50.73	
Gender, male	2,917	86.07	1,661	78.61	<0.0001
**HCC diagnosed to start sorafenib, months**					
<3	1,564	46.15	1,181	55.89	<0.0001
3–6	476	14.05	201	9.51	
6–12	521	15.37	253	11.97	
>12	828	24.43	478	22.62	
**Duration of sorafenib used, months**					
<2	1,703	50.25	1138	53.86	0.0787
2–4	692	20.42	400	18.93	
4–6	301	8.88	176	8.33	
>6	693	20.45	399	18.88	
**Dosage of sorafenib**					
<240	173	5.1	152	7.19	<0.0001
240–480	1,136	33.52	813	38.48	
480–720	259	7.64	168	7.95	
>720	1,821	53.73	980	46.38	
**Comorbidities**					
Alcoholic liver disease	197	5.81	168	7.95	0.0019
Liver cirrhosis	1,184	34.94	547	25.89	<0.0001
Liver decompensation	197	5.81	125	5.92	0.8744
Diabetes mellitus	922	27.21	873	41.32	<0.0001

* *P-value was estimated using Pearson's chi-square test*.

**Table 2:** Risk of 1-year mortality in HCC patients receiving sorafenib with or without HBV/HCV.

**Characteristic**	**Adjusted HR^a^ (95% CI)**				
	**Overall**	**Duration of sorafenib used, months**			
		** <2**	**2–4**	**4–6**	**>6**
HBV diagnosed (with HBV alone or both HBV/HCV)	1.10 (1.03–1.19)^*^	1.04 (0.95–1.14)	1.21 (1.04–1.41)^*^	1.29 (0.99–1.68)	1.27 (0.92–1.75)
No HBV and HCV diagnosed	Ref.	Ref.	Ref.	Ref.	Ref.

*HR, hazard ratio; CI, confidence interval*.

a *Adjusted for age groups, gender, HCC diagnosed to start sorafenib, dosage of sorafenib, comorbidities, and additional therapy after sorafenib*.

* *P <0.05*.

**Table 3:** Risk of follow-up period mortality in HCC patients receiving sorafenib with or without HBV/HCV.

**Characteristic**	**Adjusted HR^a^ (95% CI)**				
	**Overall**	**Follow-up period, months**			
		** <2**	**2–4**	**4–6**	**6–12**
HBV diagnosed (with HBV alone or both HBV/HCV)	1.10 (1.03–1.19)^*^	1.03 (0.90–1.19)	1.02 (0.89–1.17)	1.06 (0.90–1.25)	1.30 (1.14–1.49)^*^
No HBV and HCV diagnosed	Ref.	Ref.	Ref.	Ref.	Ref.

*HR, hazard ratio; CI, confidence interval*.

a *Adjusted for age groups, gender, HCC diagnosed to start sorafenib, dosage of sorafenib, comorbidities, and additional therapy after sorafenib*.

* *P < 0.05*.

### High EGFR Activity and Low AMPK Activity Determine the Primary Resistance of Hep3B Cells to Sorafenib

**Figure 2:**
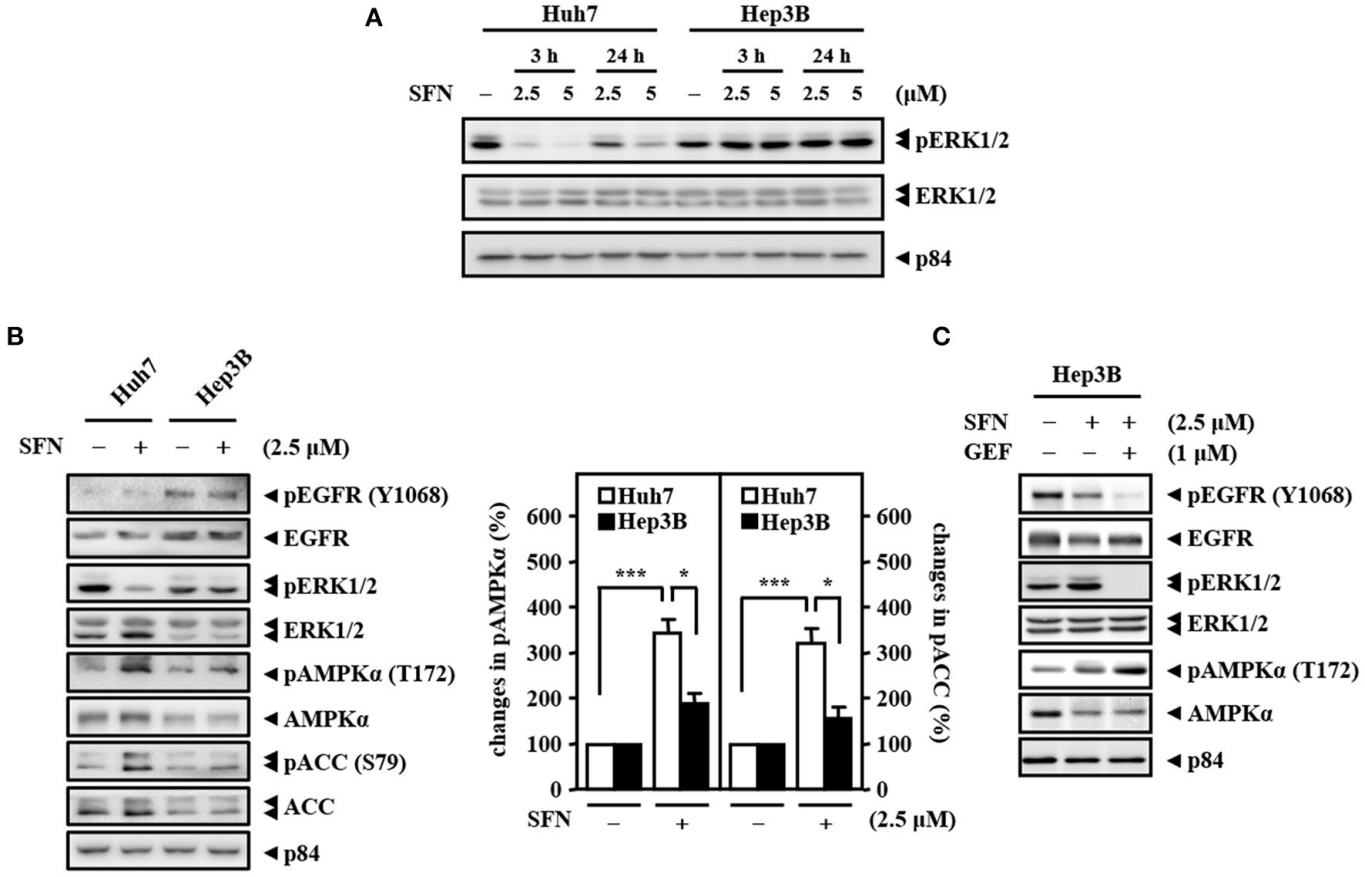
High activity of EGFR and low activity of AMPK determine the primary resistance of Hep3B cells to sorafenib. **(A)** Huh7 and Hep3B cells were treated with sorafenib at the indicated concentrations for 3 and 24 h. Whole cell lysates were harvested for Western blot analyses. **(B)** Two types of liver cancer cells (Huh7 and Hep3B) were treated with or without sorafenib (2.5 μM) for 24 h and harvested for Western blot analyses. The quantitative analyses of phosphorylated AMPK and ACC were shown in the graphs. **(C)** Hep3B cells were treated with sorafenib (2.5 μM) alone or with the combination of sorafenib and the EGFR inhibitor gefitinib (GEF; 1 μM) for 24 h. Whole cell lysates were harvested for Western blot analyses. The data are shown as the mean ± SD. *P < 0.05; **P < 0.01; ***P < 0.001 by Student's *t*-test.

To check the efficacy of sorafenib, the Raf downstream effector extracellular-signal-regulated kinase 1/2 (ERK1/2), a potential biomarker for sorafenib response, was examined in Huh7 (*HBV*-negative) and Hep3B (derived from *HBV*-infected liver) cells. The results showed that, in contrast to that in Huh7 cells, the activity of ERK1/2 (phosphorylated ERK1/2, pERK1/2) was sustainedly activated in Hep3B cells, and there was no further effect following sorafenib treatment (Figure 2A). HBV-encoded X protein (HBx) has been suggested to increase EGFR expression by inhibiting miR129-5p function (Ochi et al., 2020). Here, we found that upregulation of EGFR in *HBV*-infected liver tissues compared with healthy liver tissues through analysis of the public dataset GSE83148 (Supplementary Figure 1A). To further dissect whether EGFR contributes to sorafenib resistance in Hep3B cells, the activity of EGFR (phosphorylated EGFR, pEGFR) was examined. Western blot analyses revealed that the basal levels of EGFR and pEGFR were higher in Hep3B cells than in Huh7 cells (Figure 2B) and that the EGFR inhibitor gefitinib increased the efficacy of sorafenib by reducing the level of the phosphorylated ERK1/2 protein in Hep3B cells (Figure 2C). The above results suggest that the EGFR/ERK pathway may be linked to HBV-induced sorafenib resistance. Previous studies have indicated that ERK1/2 can promote the uncoupling of liver kinase B1 (LKB1) and AMPK to confer anti-apoptotic effects (Esteve-Puig et al., 2009). Here, we showed that the levels of phosphorylated AMPK and its downstream target acetyl-CoA carboxylase (ACC) were lower in Hep3B cells than in Huh7 cells under sorafenib treatment (Figure 2B) and that the EGFR inhibitor gefitinib strengthened sorafenib-induced AMPK phosphorylation in Hep3B cells (Figure 2C). Collectively, these results imply that the EGFR/ERK-induced reduction in AMPK phosphorylation plays a functional role in hepatocarcinoma resistance to sorafenib.

### Resistance of Hep3B Cells to Sorafenib Is Associated With Lower Autophagic Responsiveness

**Figure 3:**
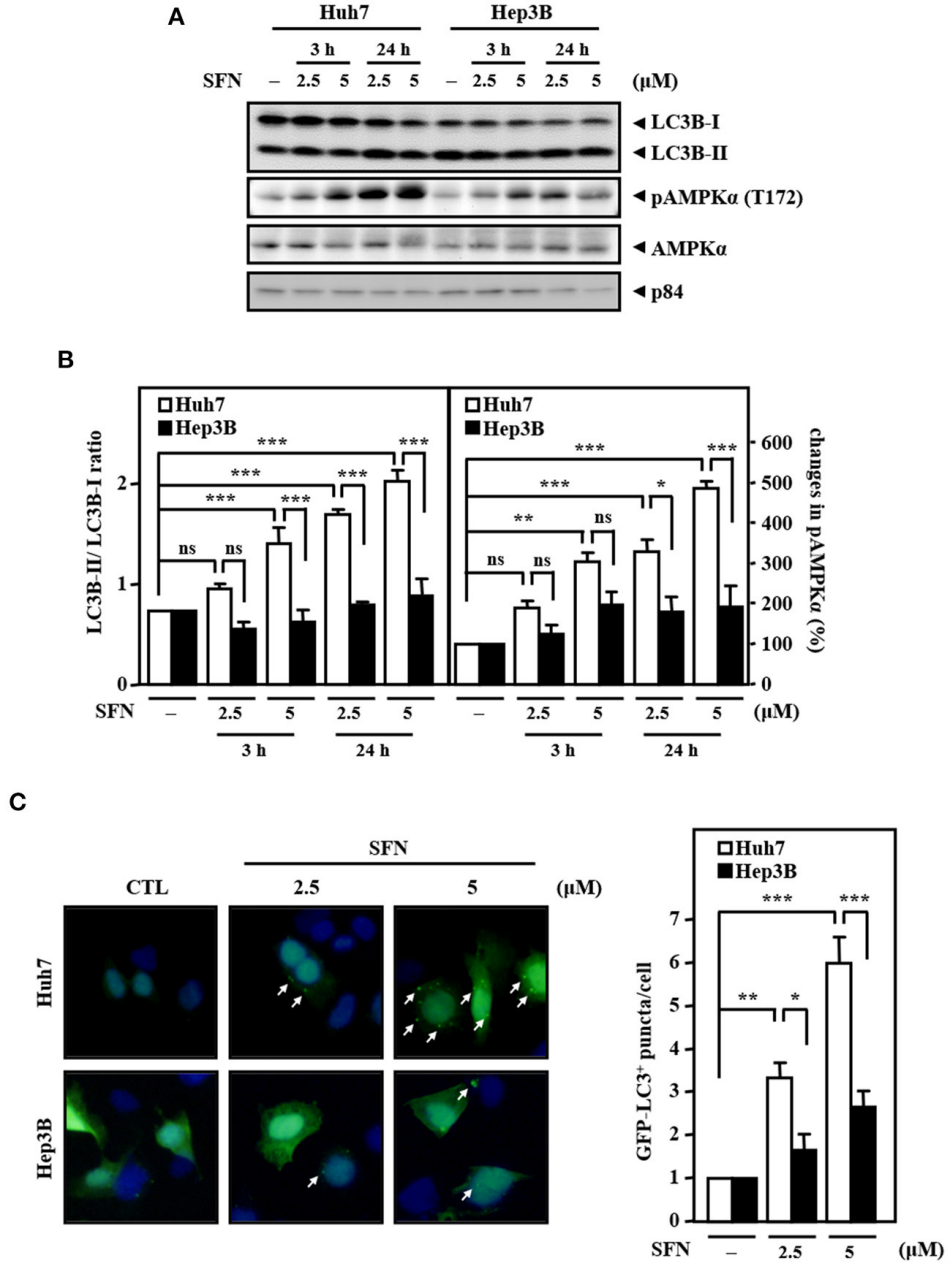
Resistance of Hep3B cells to sorafenib is associated with lower autophagic responsiveness. **(A)** Huh7 and Hep3B cells were treated with sorafenib at the indicated concentrations for 3 and 24 h. Whole cell lysates were harvested for Western blot analyses. **(B)** The quantitative analyses of phosphorylated AMPK and LC3B-II/LC3B-I were shown in the graphs. **(C)** Huh7 and Hep3B cells were transfected with GFP-LC3B expression vectors and then treated with sorafenib at the indicated concentrations for 6 h. The number of LC3B puncta was evaluated under a fluorescence microscope. The data are shown as the mean ± SD. *P < 0.05; **P < 0.01; ***P < 0.001 by Student's *t*-test.

Accumulated results have suggested that AMPK is an upstream activator of autophagy. Meanwhile, autophagy can serve as a tumor suppressor, and its deficiency leads to HCC (Liang et al., 2006; Takamura et al., 2011). However, whether AMPK and autophagy are involved in primary resistance to sorafenib in liver cancer cells remains unknown. Our results demonstrated that the levels of AMPK phosphorylation and the LC3B-II/LC3B-I ratio were significantly higher in Huh7 cells than in Hep3B cells upon sorafenib treatment (Figures 3A,B). In addition, sorafenib significantly increased the number of LC3B puncta in GFP-LC3B/Huh7 cells, but the number of LC3B puncta was marginally higher in GFP-LC3B/Hep3B cells (Figure 3C). The results suggest that the sorafenib resistance in liver cancer cells with EGFR overexpression is associated with insufficient autophagic activation.

### CEBPD Is Involved in Sorafenib-Induced Autophagic Cell Death

**Figure 4:**
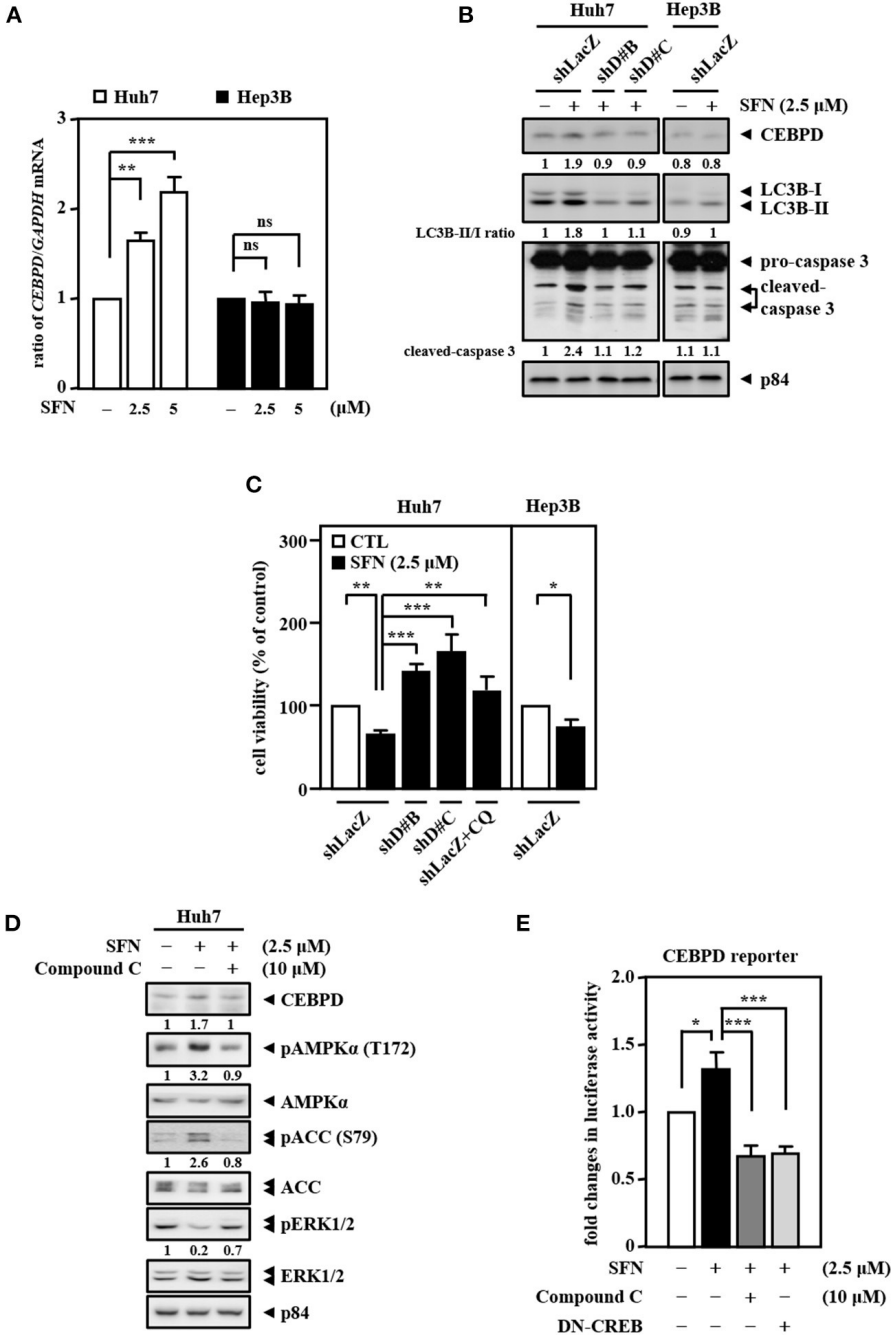
CEBPD is involved in sorafenib-induced autophagic cell death. **(A)** Huh7 and Hep3B cells were treated with sorafenib at the indicated concentrations for 3 and 24 h. Total RNA was harvested for RT-qPCR assays. **(B)** Huh7 and Hep3B cells were infected with lentiviruses encoding shLacZ (shZ) or shCEBPD (shDB and shDC) and then treated with or without sorafenib (2.5 μM). Whole cell lysates were harvested for Western blot analyses. **(C)** Huh7 and Hep3B cells were infected with lentiviruses encoding shLacZ (shZ) or shCEBPD (shDB or shDC) for 3 days. Huh7 cells were pretreated with or without chloroquine (CQ, 10 μM) for 30 min. After being treated with sorafenib (2.5 μM) for 48 h, the cell viability of the infected experimental cells was measured by MTT assays. **(D)** Huh7 cells were pretreated with or without the AMPK inhibitor compound C (10 μM) for 0.5 h and then treated with or without sorafenib (2.5 μM) for an additional 6 h. Whole cell lysates were harvested for Western blot analyses. **(E)** Huh7 cells transfected with *CEBPD* reporters were cotransfected with or without DN-CREB expression vectors for 18 h or treated with or without the AMPK inhibitor compound C (10 μM) for 30 min and then treated with or without sorafenib (2.5 μM) for an additional 3 h. The lysates of the transfected cells were harvested for luciferase assays. The data are shown as the mean ± SD. *P < 0.05; **P < 0.01; ***P < 0.001 by Student's *t*-test.

Our previous results showed that CEBPD expression is responsive to clinical anti-cancer drugs in liver cancer cells (Li et al., 2015). Here, we found that co-downregulation of CEBPD and LC3B in EGFR^high^ *HBV*-infected liver tissues compared with EGFR^low^ *HBV*-infected liver tissues through analysis of the public dataset GSE83148 (Supplementary Figure 1B). We further validated the liver specimens from the HBx transgenic mice by immunofluorescence. Consistently, we found that CEBPD and LC3B expressions were lower in tumors (T) compared with adjacent non-tumor (N) tissues (Supplementary Figure 2). Interestingly, we found that sorafenib can activate CEBPD expression in Huh7 cells but not in Hep3B cells (Figures 4A,B). In addition, the LC3B-II/LC3B-I ratio and the level of caspase-3 activation were significantly lower and the cell viability inhibition effect was minor in Hep3B cells than in Huh7 cells upon sorafenib treatment (Figures 4B,C). To verify whether CEBPD is involved in the sorafenib-induced anti-cancer effect, a loss-of-function assay was conducted by reducing the levels of CEBPD with shRNA. The results showed that the loss of CEBPD attenuated the sorafenib-induced increase in LC3B-I/-II conversion and caspase-3 activity (Figure 4B) and suppressed the sorafenib-induced inhibition in cell viability (Figure 4C) in Huh7 cells. Treatment with an autophagy inhibitor (chloroquine, CQ) also restored sorafenib-inhibited Huh7 cell viability (Figure 4C). Our previous studies suggested that the methylation status of the *CEBPD* promoter determines CEBPD induction and expression in HCC and other cancer types (Ko et al., 2008; Li et al., 2015; Chu et al., 2017). However, the methylation states of the *CEBPD* promoter were not different between Huh7 and Hep3B cells (Supplementary Figure 3), indicating that a non-DNA methylation mechanism contributes to CEBPD desensitization in liver cancer cells. AMPK is involved in CEBPD activation (Tsai et al., 2017), and the p38 MAPK/cAMP-responsive element binding protein (CREB) pathway is important for the transcriptional activation of the *CEBPD* gene (Hsiao et al., 2013; Lai et al., 2017). In addition, activation of AMPK has been reported to activate CREB in liver cancer cells (Irungbam et al., 2020). We next tested whether AMPK contributes to sorafenib-induced CEBPD expression. The results demonstrated that the AMPK inhibitor compound C suppressed sorafenib-induced AMPK and ACC phosphorylation as well as CEBPD expression in Huh7 cells (Figure 4D). Moreover, sorafenib-induced *CEBPD* reporter activity was attenuated in compound C-treated and dominant negative CREB (DN-CREB)-transfected Huh7 cells (Figure 4E). Taken together, these results suggest that AMPK participates in sorafenib-induced CEBPD expression, which contributes to autophagic cell death in HCC.

### Metformin Improves the Sensitivity of Hep3B Cells to Sorafenib

**Figure 5:**
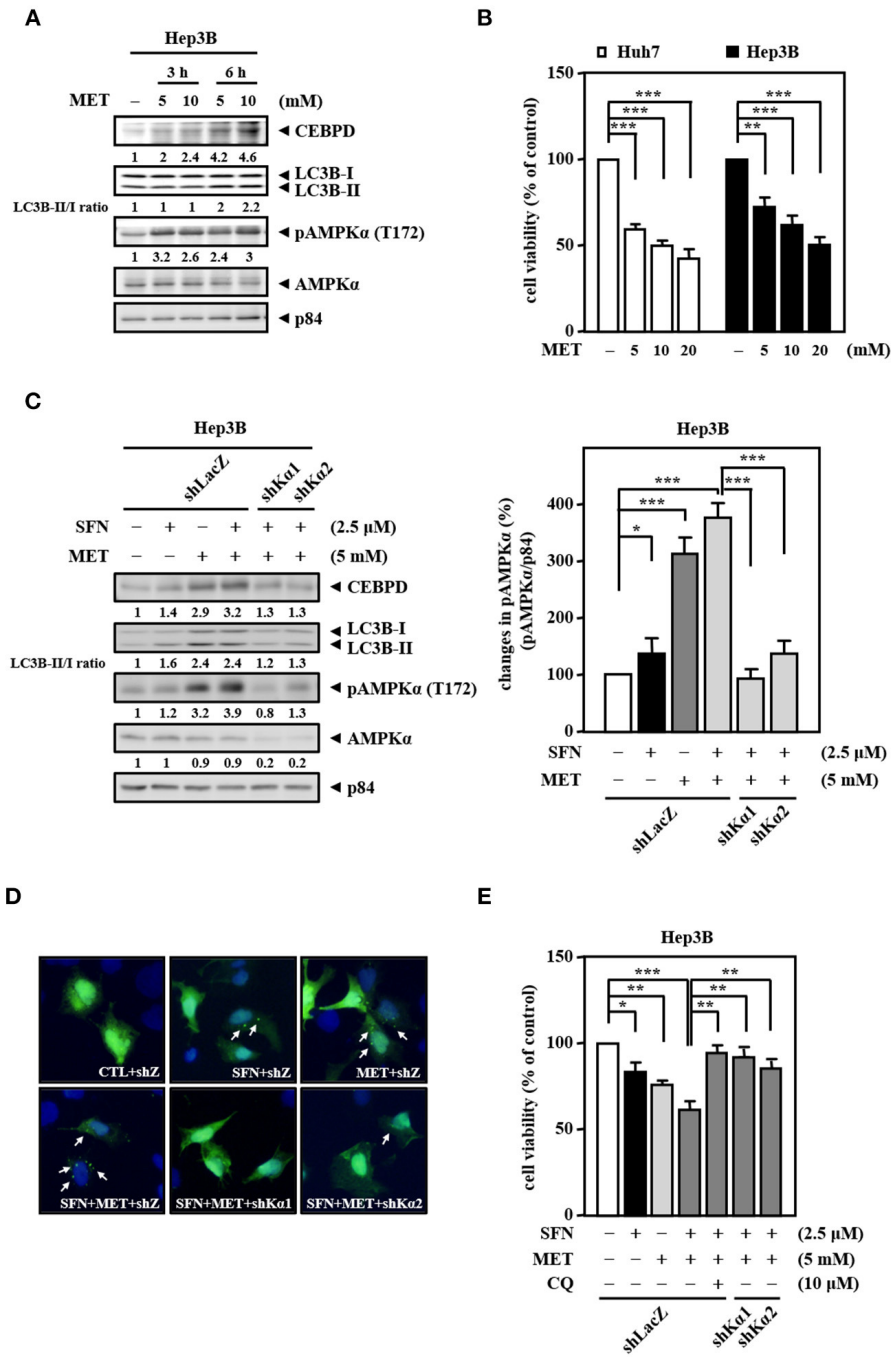
Metformin improves the sensitivity of Hep3B cells to sorafenib. **(A)** Hep3B cells were treated with metformin at the indicated concentrations for 3 and 6 h. Whole cell lysates were harvested for Western blot analyses. **(B)** Huh7 and Hep3B cells were treated with metformin (MET) at the indicated concentrations for 48 h. The cell viability of the experimental cells was measured by MTT assays after 48 h of metformin treatment at the indicated concentrations. **(C)** Hep3B cells were infected with lentiviruses encoding shLacZ (shZ) or shAMPKα (shKα1 or shKα2) for 3 days. Hep3B cells were treated with sorafenib (2.5 μM) or metformin (5 mM) alone or with the combination of sorafenib (2.5 μM) and metformin (5 mM) for 24 h. Whole cell lysates were harvested for Western blot analyses. The quantitative analysis of phosphorylated AMPK was shown in the graph. **(D)** Hep3B cells were transfected with GFP-LC3B expression vectors and then treated with sorafenib or metformin alone or with the combination of sorafenib and metformin for 6 h. The number of LC3B puncta was evaluated under a fluorescence microscope. **(E)** Hep3B cells were pretreated with or without chloroquine (CQ, 10 μM) for 30 min and then treated with sorafenib or metformin alone or with the combination of sorafenib and metformin for 48 h. The cell viability of the experimental cells was measured by MTT assays. The data are shown as the mean ± SD. *P < 0.05; **P < 0.01; ***P < 0.001 by Student's *t*-test.

Our current results suggest that sorafenib cannot efficiently induce AMPK activation to contribute to autophagic cell death due to EGFR overexpression. Since clinical drug metformin can activate AMPK bypassing the inhibitory effect of the EGFR/ERK pathway, we further assess the activity of AMPK and CEBPD in response to metformin for sorafenib resensitization. We first examined the effect of metformin on the activity of AMPK in Hep3B cells. The results revealed that metformin increased AMPK phosphorylation, CEBPD expression, and the LC3B-II/LC3B-I ratio in Hep3B cells (Figure 5A). Next, we tested whether metformin inhibits Hep3B and Huh7 cell proliferation. The results revealed that metformin reduced Huh7 and Hep3B cell viability (Figure 5B). Moreover, a combination of sorafenib and metformin was used to assess whether metformin can enhance sorafenib sensitivity in Hep3B cells. The results revealed that, compared to sorafenib treatment, combination of sorafenib and metformin significantly enhanced AMPK phosphorylation, CEBPD expression, the LC3B-II/LC3B-I ratio (Figure 5C), and the number of LC3B puncta (Figure 5D) in Hep3B cells. To further support the contribution of AMPK activity to downstream targets and biological effects, a loss-of-function assay using lentiviruses encoding shAMPKα1 and shAMPKα2 was conducted. The results showed that the knockdown of AMPKα could suppress dual treatment-induced CEBPD expression, LC3B-I/-II conversion (Figure 5C), and the number of LC3B puncta (Figure 5D) in Hep3B cells. Moreover, the combination treatment reduced Hep3B cell viability more than treatment with sorafenib or metformin alone, and either AMPKα knockdown or autophagy inhibition could also restore dual treatment-inhibited Hep3B cell viability (Figure 5E). Collectively, these results suggest that metformin can enhance the death of sorafenib-insensitive EGFR-overexpressed liver cancer cells by activating AMPK/CEBPD-induced autophagy *in vitro*.

### The Combination of Sorafenib and Metformin Elicits a Stronger Anti-tumor Effect in a Hep3B Cell Xenograft Mouse Model

**Figure 6:**
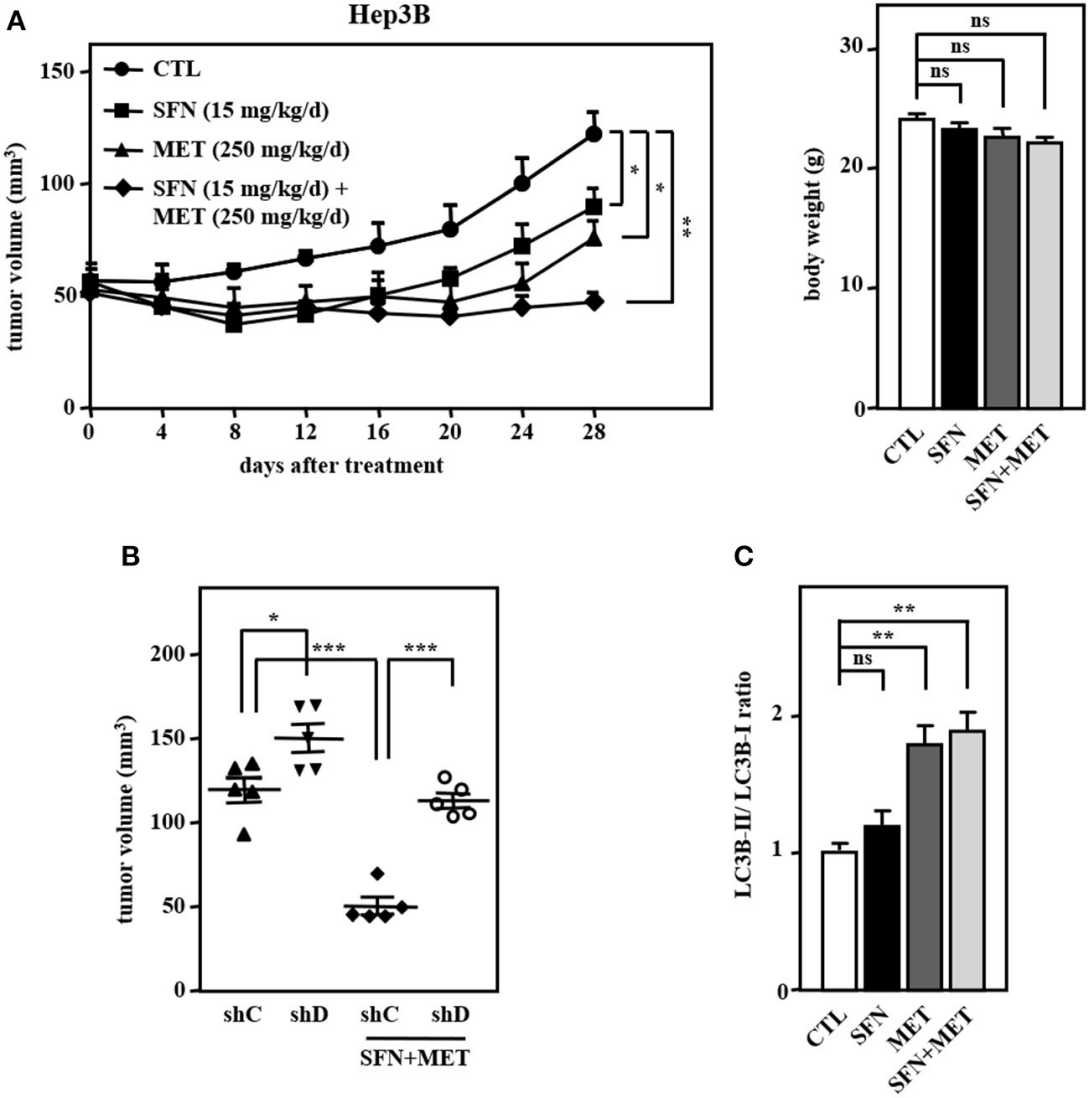
The combination of sorafenib and metformin elicits stronger cytotoxicity in a Hep3B cell xenograft mouse model. Hep3B cells were subcutaneously inoculated into NOD-SCID mice, and the mice then received an intraperitoneal injection of vehicle, sorafenib (15 mg/kg/day), metformin (250 mg/kg/day), or sorafenib (15 mg/kg/day) combined with metformin (250 mg/kg/day). **(A)** Tumor dimensions were obtained at the indicated time points. Following 4 weeks of drug treatment, the mice were sacrificed, and animal weights were obtained. **(B)** Hep3B cells were infected with lentiviruses to drive the stable expression of either IPTG-inducible LacZ shRNA (shC) or IPTG-inducible CEBPD shRNA (shD). Infected Hep3B cells were subcutaneously inoculated into the dorsum of 6-week-old NOD-SCID mice (*n* = 5), and the mice then received an intraperitoneal injection of 200 μl IPTG (0.53 mmol) every other day. The mice were then treated with or without sorafenib (15 mg/kg/day) combined with metformin (250 mg/kg/day) via intraperitoneal injection. After 28 days of treatment, the mice were sacrificed, and the tumor volume was measured. **(C)** After 28-day treatment, the lysates extracted from these experimental xenograft tumors were collected and analyzed by Western blotting. The data are shown as the mean ± SD. *P < 0.05; **P < 0.01; ***P < 0.001 by Student's *t*-test.

We further assessed the *in vivo* effect of the dual treatment of sorafenib and metformin in a human tumor xenograft mouse model in accordance with the ARRIVE guidelines (Supplementary Table 1). Concerning the effects of sorafenib dose on toxicity in HCC, we used a relatively lower dose of sorafenib in combination with metformin in Hep3B cell xenografts in NOD/SCID mice. Consistent with the above *in vitro* results, the combined treatment of sorafenib and metformin significantly enhanced cytotoxicity compared with that induced by sorafenib or metformin treatment alone (Figure 6A, left panel). Importantly, the combined treatment was well tolerated as evidenced by no weight loss was observed after treatment (Figure 6A, right panel). Furthermore, the loss of CEBPD attenuated the combined treatment-induced enhancement of Hep3B tumor xenograft death in NOD-SCID mice (Figure 6B, compare lane 1 with lane 3 and lane 3 with lane 4), suggesting that CEBPD has a strong anti-tumor effect. Importantly, the LC3B-II/LC3B-I ratio was examined in tumor lysates extracted from these experimental xenografts. The result demonstrated that the LC3B-II/LC3B-I ratio was induced in metformin treatment alone and in combination group (Figure 6C). Collectively, these results suggest that the insufficient activation of autophagy may enable residual HCC cells to survive; however, strong autophagy can contribute to cell death and resensitize sorafenib-resistant HCC cells.

## Discussion

In this study, we showed that EGFR activation is a potential determinant of the primary sorafenib resistance of HCC cells with HBV. However, the clinical results revealed that the addition of erlotinib, an oral tyrosine kinase inhibitor of EGFR with moderate anti-tumor activity against HCC, to sorafenib did not affect the overall survival (Finn, 2013). This could be partially explained by the fact that EGFR inhibitors cannot efficiently induce AMPK activation (Peng et al., 2016), and the insufficient activation of autophagy may enable residual cancer cells to resist chemotherapy. Our current results reveal that AMPK and CEBPD are unresponsive to sorafenib due to sustained EGFR/ERK activation in Hep3B cells. Therefore, metformin that has the direct effect on the activity of AMPK and CEBPD may be a potential combined with sorafenib to overcome sorafenib resistance in HCC. Interestingly, accumulation of evidence showed that metformin synergistically sensitizes leukemia cells (Wang et al., 2015) and lung cancer cells (Groenendijk et al., 2015) to sorafenib through AMPK activation, which are consistent with our findings.

There are other downstream signaling pathways regulated by EGFR, including Src/signal transducer and activator of transcription (STAT) and phosphatidylinositol 3-kinase (PI3K)/Akt signal transduction pathways (Nyati et al., 2006). Our previous finding revealed that metformin can reduce Src-mediated CEBPD protein degradation (Tsai et al., 2017). However, Western blot analyses revealed that sorafenib activates Src. Meanwhile, the AMPK inhibitor compound C has no effect on sorafenib-induced Src phosphorylation (Supplementary Figure 4). These results suggest that metformin could work via the Src-dependent pathway to enhance CEBPD expression and autophagic cell death in sorafenib-resistant liver cancer cells. Moreover, several studies have demonstrated that metformin inhibits STAT (Feng et al., 2014) and the PI3K/Akt pathway (Pernicova and Korbonits, 2014). Therefore, the application of metformin for the improvement of the efficacy of sorafenib in HCC with EGFR overexpression involves multiple factors that need to be further investigated.

Acute inflammation is a strong and rapid response to tissue injury and protects body, but low-grade and chronic inflammation can be harmful. Sustained cell growth in an inflammatory environment combined with accumulation of genetic abnormalities contributes to cancer progression. Our previous study demonstrated that inflammation-responsive transcription factor CEBPD can induce genomic instability and promote tumorigenesis, even though it serves as a tumor suppressor in cervical cancer (Wu et al., 2011). Recent study indicated that the acquired sorafenib resistance may also be associated with genomic instability (Xia et al., 2020). Therefore, the association of insufficient but sustained CEBPD and autophagic activity with genomic instability and cell survival, respectively, in response to sorafenib deserves to be clarified. In this way, the molecular details of how sorafenib establishes acquired resistance will be dissected. The dual roles of CEBPD may orchestrate the dual functions of autophagy to contribute to both death and resistance of cancer cells and this could in part underlie the complex role of inflammation in cancer development.

We propose that metformin, an AMPK activator, restores the sensitivity of EGFR-overexpressed liver cancer cells to sorafenib. However, many anti-diabetic drugs and small molecule compounds should be tested with the goal of activating AMPK in cancer cells. Insulin-sensitizing thiazolidinediones (TZDs) are potent agonist ligands for the nuclear hormone receptor peroxisome proliferator-activated receptor γ (PPARγ). They are also thought to exert some of their anti-diabetic effects through AMPK activation in a variety of tissues, including skeletal muscle (LeBrasseur et al., 2006) and liver (Saha et al., 2004). Glucagon-like peptide-1 (GLP-1) mimetics stimulate insulin secretion in a glucose-dependent manner. Previous studies have shown that these compounds and endogenous GLP-1 can activate the AMPK pathway (Svegliati-Baroni et al., 2011). The first direct AMPK activator was A-769662, but this compound is unsuitable to be used due to its poor oral absorption. Recently, compound 991 is significantly more potent than A-769662 in allosterically activating AMPK (Xiao et al., 2013). Although further clinical trials are needed to evaluate the safety and efficacy of these compounds, our results indicate that therapeutic AMPK activation should be an attractive target for improving the efficacy of sorafenib.

## Conclusions

**Figure 7:**
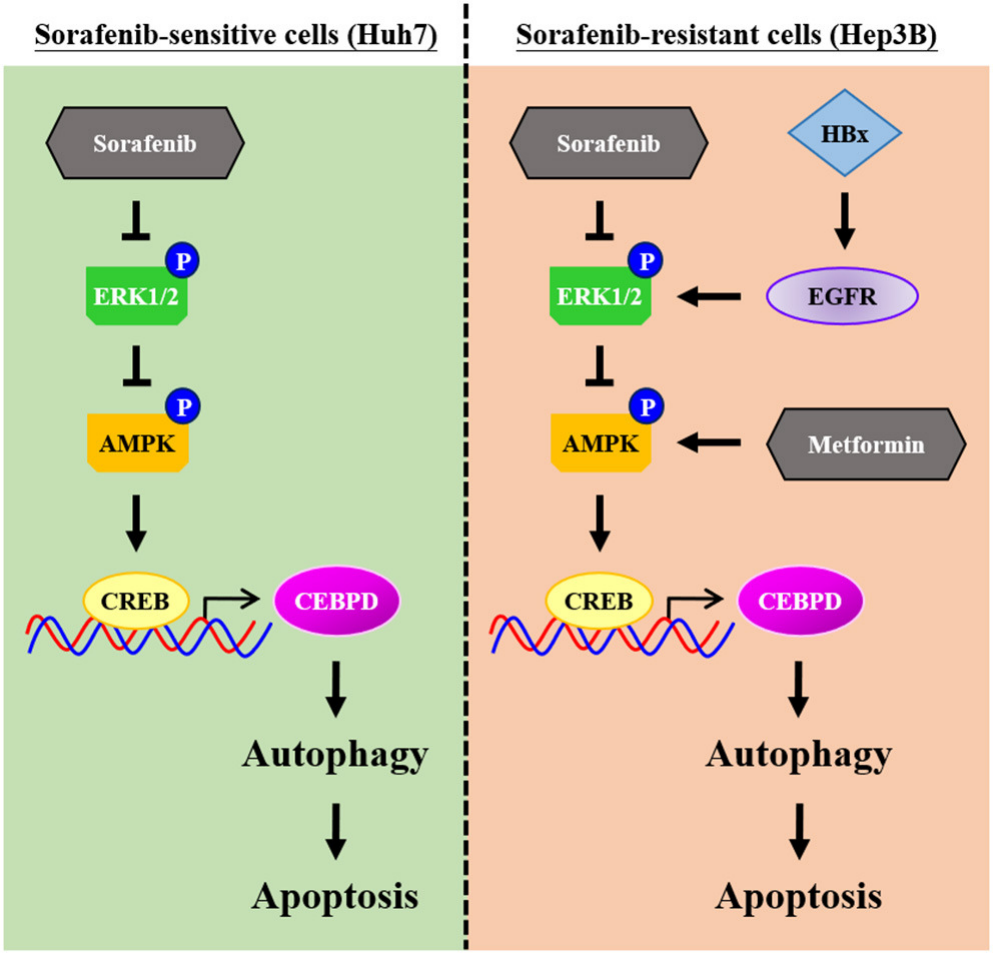
Schematic model of the molecular mechanism by which metformin increases the sensitivity of Hep3B cells to sorafenib. Hep3B cells are intrinsically more resistant than Huh7 cells to sorafenib. High activity of epidermal growth factor receptor (EGFR) and low autophagic responsiveness may determine the primary resistance of Hep3B cells to sorafenib. CCAAT/enhancer binding protein delta (CEBPD), a potent tumor suppressor, is responsive to metformin via AMP-activated protein kinase (AMPK) activation, and it promotes autophagic cell death. Furthermore, metformin can resensitize sorafenib-induced autophagic cell death in Hep3B cells. Taken together, our results provide possible implications for improving the efficacy of sorafenib and helping to develop personalized medicine strategies for HCC patients.

Even though new chemotherapy agents are being developed quickly, all chemotherapy agents face the challenge of drug resistance. Primary drug resistance is one of the reasons for the attenuation of the efficacy of chemotherapy agents. In the current study, our results revealed a new insight that insufficient AMPK and CEBPD activation as well as lower autophagic activity play a functional role in sorafenib resistance in liver cancer cells with EGFR overexpression. Meanwhile, we further demonstrated that metformin may be combined with sorafenib to strengthen autophagic cell death (Figure 7). The discoveries indicated that AMPK activators and autophagy activators could be potential candidates for further application in sorafenib-resistant liver cancers.

## Data Availability Statement

The dataset analysed during the current study is available in the public dataset GSE83148 from GEO (National Center for Biotechnology Information, Bethesda, MD).

## Ethics Statement

The studies involving human participants were reviewed and approved by Institutional Review Board of Chi-Mei Medical Center in Taiwan (IRB: 10702-E04). Written informed consent for participation was not required for this study in accordance with the national legislation and the institutional requirements. The animal study was reviewed and approved by Institutional Animal Care and Use Committee (IACUC) of College of Medicine, National Cheng Kung University (Approval Number: 103209).

## Author Contributions

H-YLa, H-HT, C-FL, and J-MW: conceptualization. H-YLa, H-HT, C-JY, L-YH, C-CY, C-HH, H-YLi, and F-WC: methodology and formal analysis. H-YLa, H-HT, C-JY, L-YH, C-CY, and C-HH: investigation. H-YLa, H-HT, C-JY, L-YH, C-CY, C-HH, C-FL, and J-MW: resources. H-YLa and H-HT: validation, visualization, and writing–original draft. H-YLa and J-MW: writing–review & editing. J-MW: funding acquisition and supervision. All authors contributed to the article and approved the submitted version.

## Conflict of Interest

The authors declare that the research was conducted in the absence of any commercial or financial relationships that could be construed as a potential conflict of interest.

## Supplementary Material

Supplementary Figure 1 EGFR upregulation is associated with lower CEBPD and LC3B levels in *HBV*-infected livers. **(A)** Quantitative data of *EGFR* transcripts were extracted from the transcript microarray of 6 healthy livers and 122 *HBV*-infected livers. **(B)** *HBV*-infected liver tissues were divided into two groups according to the median value of EGFR levels (EGFR^low^ and EGFR^high^). Quantitative data of *CEBPD* and *LC3B* transcripts were extracted from the transcript microarray of EGFR^low^ and EGFR^high^ *HBV*-infected livers.

Supplementary Figure 2 CEBPD and LC3B are co-downregulated in tumors of HBx transgenic mice. The liver specimens [tumors (T) and adjacent non-tumor (N) tissues] from the 18-month old HBx transgenic mice were analyzed by immunohistochemistry using CEBPD and LC3B antibodies.

Supplementary Figure 3 Methylation states on the *CEBPD* promoter are not different in Huh7 and Hep3B cells. The CpG methylation status of *CEBPD* promoters in Huh7 and Hep3B cells was determined using MSP assays.

Supplementary Figure 4 The effects of sorafenib and the AMPK inhibitor compound C on Src phosphorylation in liver cancer cells. **(A)** Two types of liver cancer cells (Huh7 and Hep3B) were treated with or without sorafenib (2.5 μM) for 24 h and harvested for Western blot analyses. **(B)** Huh7 cells were pretreated with or without the AMPK inhibitor compound C (10 μM) for 0.5 h and then treated with or without sorafenib (2.5 μM) for an additional 6 h. Whole cell lysates were harvested for Western blot analyses and examined indicated proteins with specific antibodies.

Supplementary Table 1 Animal studies comply with the ARRIVE guidelines.

## Abbreviations

AMPK: AMP-activated protein kinase
CEBPD: CCAAT/enhancer binding protein delta
EGFR: epidermal growth factor receptor
ERK: extracellular-signal-regulated kinase
HBV: hepatitis B virus
HBx: HBV-encoded X protein
HCC: hepatocellular carcinoma
LC3B: light chain 3 beta.

## References

[B1] Agrawal S. Hofmann W. K. Tidow N. Ehrich M. van den Boom D. Koschmieder S.. (2007). The C/EBPdelta tumor suppressor is silenced by hypermethylation in acute myeloid leukemia. Blood 109, 3895–3905. 10.1182/blood-2006-08-040147 17234736

[B2] Bray F. Parkin D. M. (2009). Evaluation of data quality in the cancer registry: principles and methods. Part I: comparability, validity and timeliness. Eur. J. Cancer 45, 747–755. 10.1016/j.ejca.2008.11.032 19117750

[B3] Buckley A. F. Burgart L. J. Sahai V. Kakar S. (2008). Epidermal growth factor receptor expression and gene copy number in conventional hepatocellular carcinoma. Am. J. Clin. Pathol. 129, 245–251. 10.1309/WF10QAAED3PP93BH 18208805

[B4] Cervello M. McCubrey J. A. Cusimano A. Lampiasi N. Azzolina A. Montalto G. (2012). Targeted therapy for hepatocellular carcinoma: novel agents on the horizon. Oncotarget 3, 236–260. 10.18632/oncotarget.466 22470194 PMC3359882

[B5] Chan K. M. Wu T. H. Cheng C. H. Lee C. F. Wu T. J. Chou H. S.. (2019). Implementation of sorafenib treatment for advanced hepatocellular carcinoma: an illustration of current practice in Taiwan. Cancer Manag. Res. 11, 1013–1021. 10.2147/CMAR.S186678 30774429 PMC6349081

[B6] Chang L. H. Huang H. S. Wu P. T. Jou I. M. Pan M. H. Chang W. C.. (2012). Role of macrophage CCAAT/enhancer binding protein delta in the pathogenesis of rheumatoid arthritis in collagen-induced arthritic mice. PLoS ONE 7:e45378. 10.1371/journal.pone.0045378 23028973 PMC3454428

[B7] Chen H. P. Shieh J. J. Chang C. C. Chen T. T. Lin J. T. Wu M. S.. (2013). Metformin decreases hepatocellular carcinoma risk in a dose-dependent manner: population-based and *in vitro* studies. Gut 62, 606–615. 10.1136/gutjnl-2011-301708 22773548

[B8] Chiang C. J. Lo W. C. Yang Y. W. You S. L. Chen C. J. Lai M. S. (2016). Incidence and survival of adult cancer patients in Taiwan, 2002-2012. J. Formos. Med. Assoc. 115, 1076–1088. 10.1016/j.jfma.2015.10.011 26786251

[B9] Chu Y. Y. Ko C. Y. Wang S. M. Lin P. I. Wang H. Y. Lin W. C.. (2017). Bortezomib-induced miRNAs direct epigenetic silencing of locus genes and trigger apoptosis in leukemia. Cell Death Dis. 8:e3167. 10.1038/cddis.2017.520 29120412 PMC5775404

[B10] Esteve-Puig R. Canals F. Colome N. Merlino G. Recio J. A. (2009). Uncoupling of the LKB1-AMPKalpha energy sensor pathway by growth factors and oncogenic BRAF. PLoS ONE 4:e4771. 10.1371/journal.pone.0004771 19274086 PMC2651576

[B11] Feng Y. Ke C. Tang Q. Dong H. Zheng X. Lin W.. (2014). Metformin promotes autophagy and apoptosis in esophageal squamous cell carcinoma by downregulating Stat3 signaling. Cell Death Dis. 5:e1088. 10.1038/cddis.2014.59 24577086 PMC3944271

[B12] Finn R. S. (2013). Emerging targeted strategies in advanced hepatocellular carcinoma. Semin. Liver Dis. 33(Suppl. 1), S11–S19. 10.1055/s-0033-1333632 23457035

[B13] Groenendijk F. H. Mellema W. W. van der Burg E. Schut E. Hauptmann M. Horlings H. M.. (2015). Sorafenib synergizes with metformin in NSCLC through AMPK pathway activation. Int. J. Cancer. 136, 1434–1444. 10.1002/ijc.29113 25080865 PMC4312923

[B14] Hsiao Y. W. Li C. F. Chi J. Y. Tseng J. T. Chang Y. Hsu L. J.. (2013). CCAAT/enhancer binding protein delta in macrophages contributes to immunosuppression and inhibits phagocytosis in nasopharyngeal carcinoma. Sci. Signal. 6:ra59. 10.1126/scisignal.2003648 23861541

[B15] Irungbam K. Roderfeld M. Glimm H. Hempel F. Schneider F. Hehr L.. (2020). Cholestasis impairs hepatic lipid storage via AMPK and CREB signaling in hepatitis B virus surface protein transgenic mice. Lab. Invest. 100, 1411–1424. 10.1038/s41374-020-0457-9 32612285 PMC7572243

[B16] Jackson R. Psarelli E. E. Berhane S. Khan H. Johnson P. (2017). Impact of viral status on survival in patients receiving sorafenib for advanced hepatocellular cancer: a meta-analysis of randomized phase III trials. J. Clin. Oncol. 35, 622–628. 10.1200/JCO.2016.69.5197 28045619

[B17] Kabeya Y. Mizushima N. Ueno T. Yamamoto A. Kirisako T. Noda T.. (2000). LC3, a mammalian homologue of yeast Apg8p, is localized in autophagosome membranes after processing. EMBO J. 19, 5720–5728. 10.1093/emboj/19.21.5720 11060023 PMC305793

[B18] Khan M. Imam H. Siddiqui A. (2018). Subversion of cellular autophagy during virus infection: Insights from hepatitis B and hepatitis C viruses. Liver Res. 2, 146–156. 10.1016/j.livres.2018.09.002 31803515 PMC6892584

[B19] Kim I. He Y. Y. (2013). Targeting the AMP-activated protein kinase for cancer prevention and therapy. Front. Oncol. 3:175. 10.3389/fonc.2013.00175 23875169 PMC3711071

[B20] Ko C. Y. Hsu H. C. Shen M. R. Chang W. C. Wang J. M. (2008). Epigenetic silencing of CCAAT/enhancer-binding protein delta activity by YY1/polycomb group/DNA methyltransferase complex. J. Biol. Chem. 283, 30919–30932. 10.1074/jbc.M804029200 18753137 PMC2662167

[B21] Lai H. Y. Hsu L. W. Tsai H. H. Lo Y. C. Yang S. H. Liu P. Y.. (2017). CCAAT/enhancer-binding protein delta promotes intracellular lipid accumulation in M1 macrophages of vascular lesions. Cardiovasc. Res. 113, 1376–1388. 10.1093/cvr/cvx134 28859294

[B22] LeBrasseur N. K. Kelly M. Tsao T. S. Farmer S. R. Saha A. K. Ruderman N. B.. (2006). Thiazolidinediones can rapidly activate AMP-activated protein kinase in mammalian tissues. Am. J. Physiol. Endocrinol. Metab. 291, E175–E181. 10.1152/ajpendo.00453.2005 16464908

[B23] Li C. F. Tsai H. H. Ko C. Y. Pan Y. C. Yen C. J. Lai H. Y.. (2015). HMDB and 5-AzadC combination reverses tumor suppressor CCAAT/enhancer-binding protein delta to strengthen the death of liver cancer cells. Mol. Cancer Ther. 14, 2623–2633. 10.1158/1535-7163.MCT-15-0025 26358750

[B24] Liang C. Feng P. Ku B. Dotan I. Canaani D. Oh B. H.. (2006). Autophagic and tumour suppressor activity of a novel Beclin1-binding protein UVRAG. Nat. Cell Biol. 8, 688–699. 10.1038/ncb1426 16799551

[B25] Liu Y. Levine B. (2015). Autosis and autophagic cell death: the dark side of autophagy. Cell Death Differ. 22, 367–376. 10.1038/cdd.2014.143 25257169 PMC4326571

[B26] Lu L. C. Chen P. J. Yeh Y. C. Hsu C. H. Chen H. M. Lai M. S.. (2017). Prescription Patterns of sorafenib and outcomes of patients with advanced hepatocellular carcinoma: a national population study. Anticancer Res. 37, 2593–2599. 10.21873/anticanres.11604 28476832

[B27] Nyati M. K. Morgan M. A. Feng F. Y. Lawrence T. S. (2006). Integration of EGFR inhibitors with radiochemotherapy. Nat. Rev. Cancer 6, 876–885. 10.1038/nrc1953 17036041

[B28] Ochi M. Otsuka M. Maruyama R. Koike K. (2020). HBx increases EGFR expression by inhibiting miR129-5p function. Biochem. Biophys. Res. Commun. 529, 198–203. 10.1016/j.bbrc.2020.06.018 32703411

[B29] O'Rourke J. Yuan R. DeWille J. (1997). CCAAT/enhancer-binding protein-delta (C/EBP-delta) is induced in growth-arrested mouse mammary epithelial cells. J. Biol. Chem. 272, 6291–6296. 10.1074/jbc.272.10.6291 9045647

[B30] Pan Y. C. Li C. F. Ko C. Y. Pan M. H. Chen P. J. Tseng J. T.. (2010). CEBPD reverses RB/E2F1-mediated gene repression and participates in HMDB-induced apoptosis of cancer cells. Clin. Cancer Res. 16, 5770–5780. 10.1158/1078-0432.CCR-10-1025 20971808 PMC7325841

[B31] Park M. A. Reinehr R. Haussinger D. Voelkel-Johnson C. Ogretmen B. Yacoub A.. (2010). Sorafenib activates CD95 and promotes autophagy and cell death via Src family kinases in gastrointestinal tumor cells. Mol. Cancer Ther. 9, 2220–2231. 10.1158/1535-7163.MCT-10-0274 20682655 PMC2933415

[B32] Peng M. Huang Y. Tao T. Peng C. Y. Su Q. Xu W.. (2016). Metformin and gefitinib cooperate to inhibit bladder cancer growth via both AMPK and EGFR pathways joining at Akt and Erk. Sci. Rep. 6:28611. 10.1038/srep28611 27334428 PMC4917871

[B33] Pernicova I. Korbonits M. (2014). Metformin–mode of action and clinical implications for diabetes and cancer. Nat. Rev. Endocrinol. 10, 143–156. 10.1038/nrendo.2013.256 24393785

[B34] Saha A. K. Avilucea P. R. Ye J. M. Assifi M. M. Kraegen E. W. Ruderman N. B. (2004). Pioglitazone treatment activates AMP-activated protein kinase in rat liver and adipose tissue *in vivo*. Biochem. Biophys. Res. Commun. 314, 580–585. 10.1016/j.bbrc.2003.12.120 14733947

[B35] Shi W. Y. Xiao D. Wang L. Dong L. H. Yan Z. X. Shen Z. X.. (2012). Therapeutic metformin/AMPK activation blocked lymphoma cell growth via inhibition of mTOR pathway and induction of autophagy. Cell Death Dis. 3:e275. 10.1038/cddis.2012.13 22378068 PMC3317343

[B36] Sivko G. S. DeWille J. W. (2004). CCAAT/Enhancer binding protein delta (c/EBPdelta) regulation and expression in human mammary epithelial cells: I. “Loss of function” alterations in the c/EBPdelta growth inhibitory pathway in breast cancer cell lines. J. Cell. Biochem. 93, 830–843. 10.1002/jcb.20223 15389879

[B37] Svegliati-Baroni G. Saccomanno S. Rychlicki C. Agostinelli L. De Minicis S. Candelaresi C.. (2011). Glucagon-like peptide-1 receptor activation stimulates hepatic lipid oxidation and restores hepatic signalling alteration induced by a high-fat diet in nonalcoholic steatohepatitis. Liver Int. 31, 1285–1297. 10.1111/j.1478-3231.2011.02462.x 21745271

[B38] Takamura A. Komatsu M. Hara T. Sakamoto A. Kishi C. Waguri S.. (2011). Autophagy-deficient mice develop multiple liver tumors. Genes Dev. 25, 795–800. 10.1101/gad.2016211 21498569 PMC3078705

[B39] Tomic T. Botton T. Cerezo M. Robert G. Luciano F. Puissant A.. (2011). Metformin inhibits melanoma development through autophagy and apoptosis mechanisms. Cell Death Dis. 2:e199. 10.1038/cddis.2011.86 21881601 PMC3186904

[B40] Tsai H. H. Lai H. Y. Chen Y. C. Li C. F. Huang H. S. Liu H. S.. (2017). Metformin promotes apoptosis in hepatocellular carcinoma through the CEBPD-induced autophagy pathway. Oncotarget 8, 13832–13845. 10.18632/oncotarget.14640 28099155 PMC5355142

[B41] Wang F. Liu Z. Zeng J. Zhu H. Li J. Cheng X.. (2015). Metformin synergistically sensitizes FLT3-ITD-positive acute myeloid leukemia to sorafenib by promoting mTOR-mediated apoptosis and autophagy. Leuk. Res. 39, 1421–1427. 10.1016/j.leukres.2015.09.016 26505133

[B42] Wang J. M. Tseng J. T. Chang W. C. (2005). Induction of human NF-IL6beta by epidermal growth factor is mediated through the p38 signaling pathway and cAMP response element-binding protein activation in A431 cells. Mol. Biol. Cell. 16, 3365–3376. 10.1091/mbc.e05-02-0105 15901830 PMC1165418

[B43] Wang W. J. Li C. F. Chu Y. Y. Wang Y. H. Hour T. C. Yen C. J.. (2017). Inhibition of the EGFR/STAT3/CEBPD axis reverses cisplatin cross-resistance with paclitaxel in the urothelial carcinoma of the urinary bladder. Clin. Cancer Res. 23, 503–513. 10.1158/1078-0432.CCR-15-1169 27435393

[B44] Wang X. Li W. Zhang N. Zheng X. Jing Z. (2019). Opportunities and challenges of co-targeting epidermal growth factor receptor and autophagy signaling in non-small cell lung cancer. Oncol. Lett. 18, 499–506. 10.3892/ol.2019.10372 31289521 PMC6546992

[B45] Wang Z. Han W. Sui X. Fang Y. Pan H. (2014). Autophagy: a novel therapeutic target for hepatocarcinoma (review). Oncol. Lett. 7, 1345–1351. 10.3892/ol.2014.1916 24765136 PMC3997714

[B46] Wu D. H. Jia C. C. Chen J. Lin Z. X. Ruan D. Y. Li X.. (2014). Autophagic LC3B overexpression correlates with malignant progression and predicts a poor prognosis in hepatocellular carcinoma. Tumour Biol. 35, 12225–12233. 10.1007/s13277-014-2531-7 25256671

[B47] Wu S. R. Li C. F. Hung L. Y. Huang A. M. Tseng J. T. Tsou J. H.. (2011). CCAAT/enhancer-binding protein delta mediates tumor necrosis factor alpha-induced Aurora kinase C transcription and promotes genomic instability. J. Biol. Chem. 286, 28662–28670. 10.1074/jbc.M111.270710 21715338 PMC3190673

[B48] Xia S. Pan Y. Liang Y. Xu J. Cai X. (2020). The microenvironmental and metabolic aspects of sorafenib resistance in hepatocellular carcinoma. EBioMedicine 51:102610. 10.1016/j.ebiom.2019.102610 31918403 PMC7000339

[B49] Xiao B. Sanders M. J. Carmena D. Bright N. J. Haire L. F. Underwood E.. (2013). Structural basis of AMPK regulation by small molecule activators. Nat. Commun. 4:3017. 10.1038/ncomms4017 24352254 PMC3905731

[B50] Zhu Y. J. Zheng B. Wang H. Y. Chen L. (2017). New knowledge of the mechanisms of sorafenib resistance in liver cancer. Acta Pharmacol. Sin. 38, 614–622. 10.1038/aps.2017.5 28344323 PMC5457690

