# Supplementary figures and images for: Metformin Resensitizes Sorafenib-Resistant HCC Cells Through AMPK-Dependent Autophagy Activation
Source: Front Cell Dev Biol. 2021 Jan 21;8:596655. doi: 10.3389/fcell.2020.596655 (PMC7931828; doi:10.3389/fcell.2020.596655)

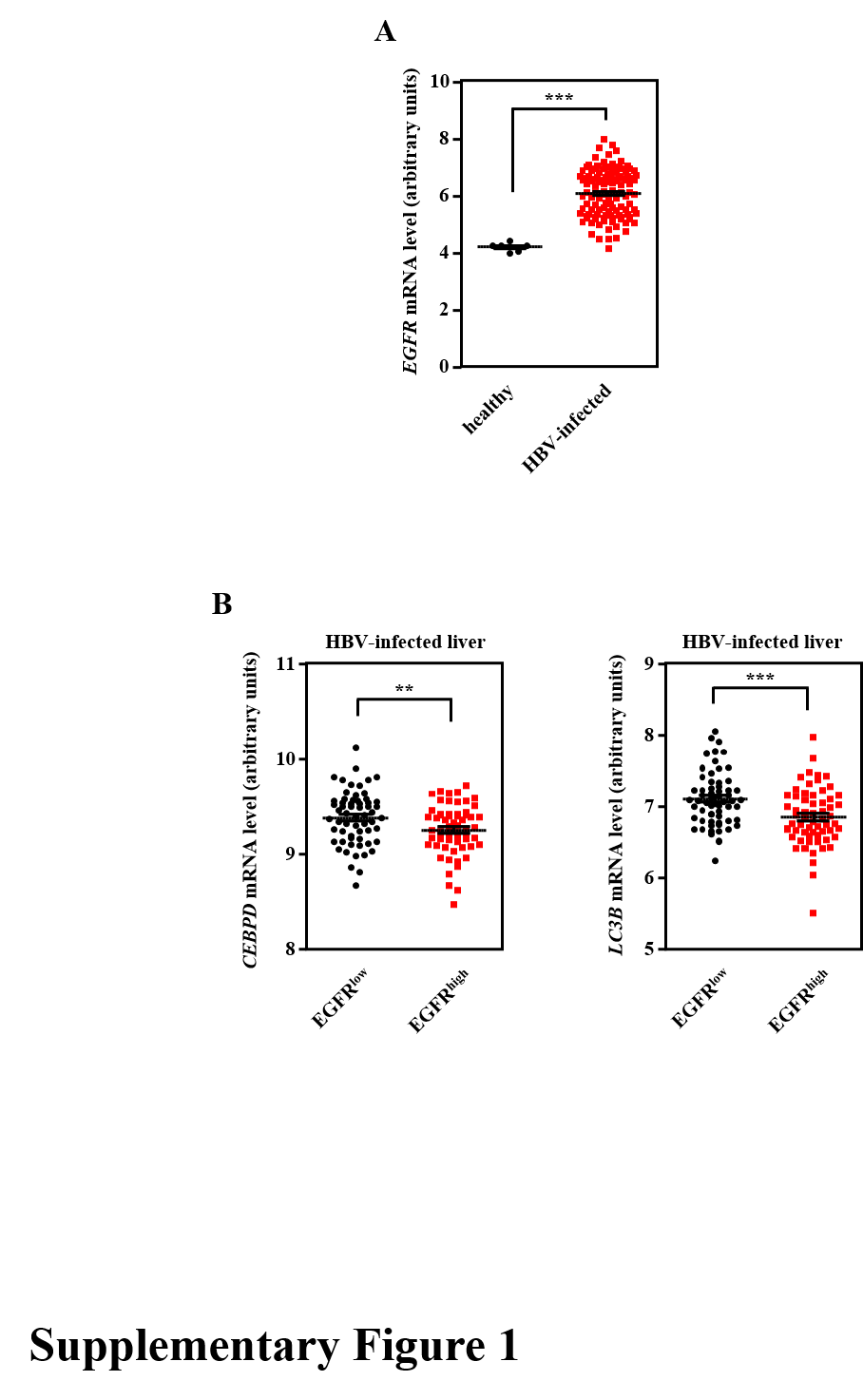

Supplement: Supplementary Figure 1 — EGFR upregulation is associated with lower CEBPD and LC3B levels in HBV-infected livers. (A) Quantitative data of EGFR transcripts were extracted from the transcript microarray of 6 healthy livers and 122 HBV-infected livers. (B) HBV-infected liver tissues were divided into two groups according to the median value of EGFR levels (EGFRlow and EGFRhigh). Quantitative data of CEBPD and LC3B transcripts were extracted from the transcript microarray of EGFRlow and EGFRhigh HBV-infected livers. [file Figure_1.TIF]

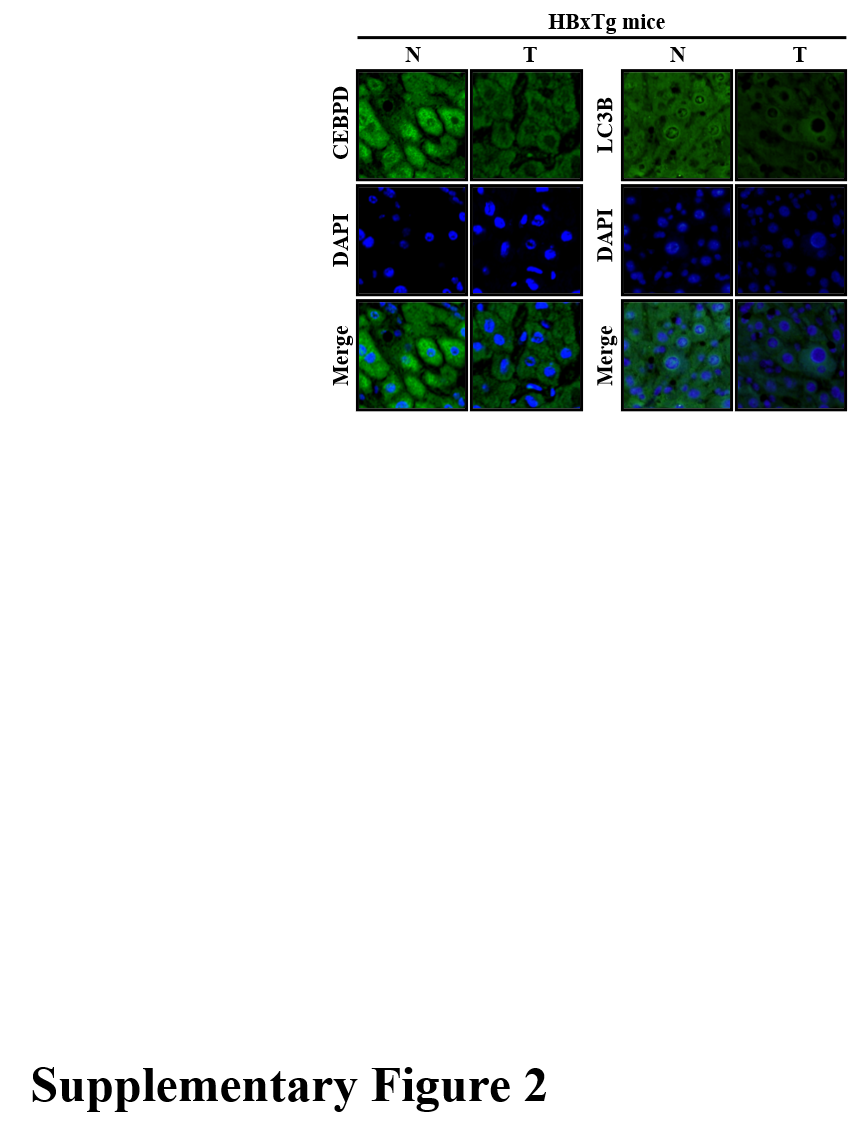

Supplement: Supplementary Figure 2 — CEBPD and LC3B are co-downregulated in tumors of HBx transgenic mice. The liver specimens [tumors (T) and adjacent non-tumor (N) tissues] from the 18-month old HBx transgenic mice were analyzed by immunohistochemistry using CEBPD and LC3B antibodies. [file Figure_2.TIF]

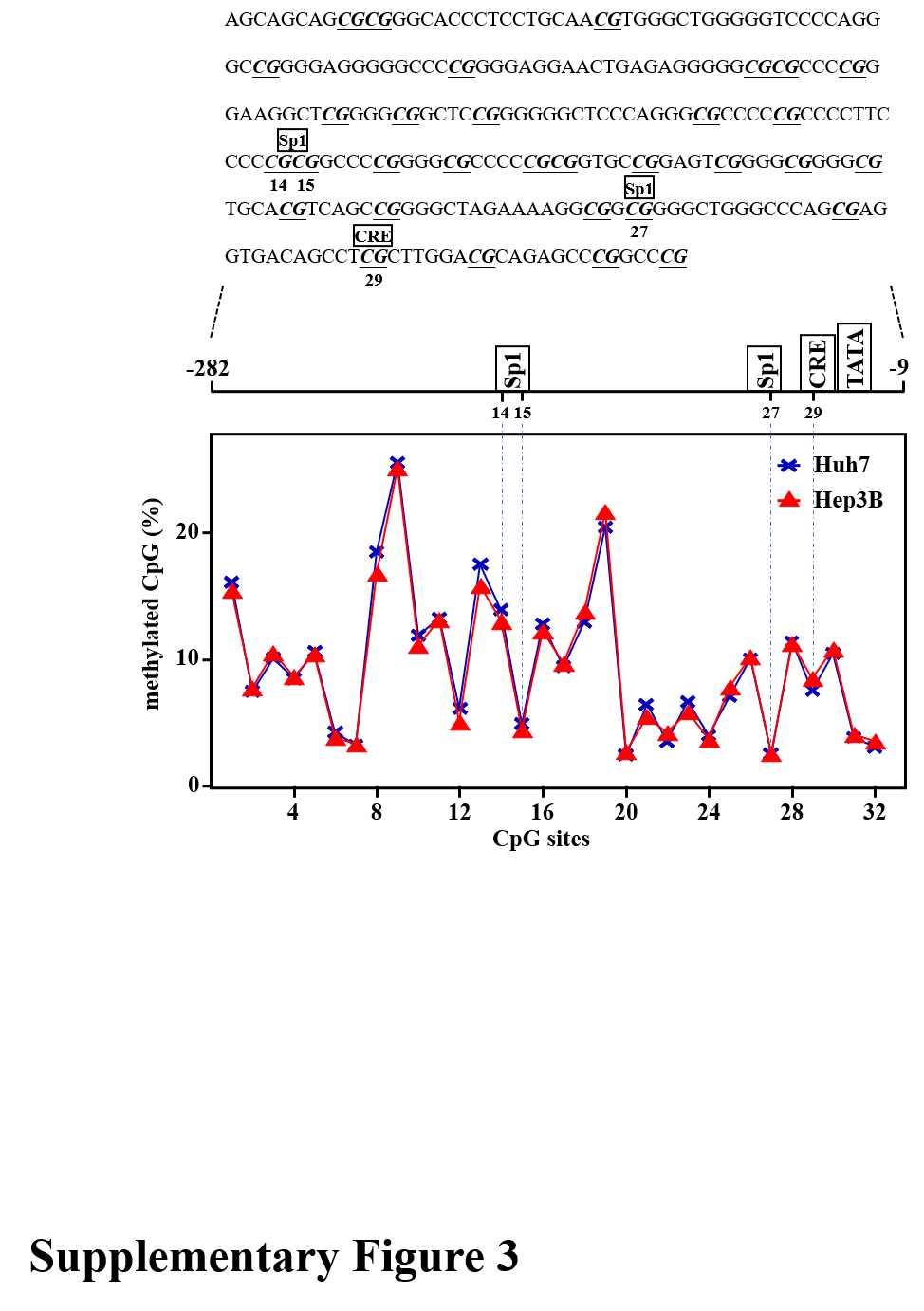

Supplement: Supplementary Figure 3 — Methylation states on the CEBPD promoter are not different in Huh7 and Hep3B cells. The CpG methylation status of CEBPD promoters in Huh7 and Hep3B cells was determined using MSP assays. [file Figure_3.TIF]

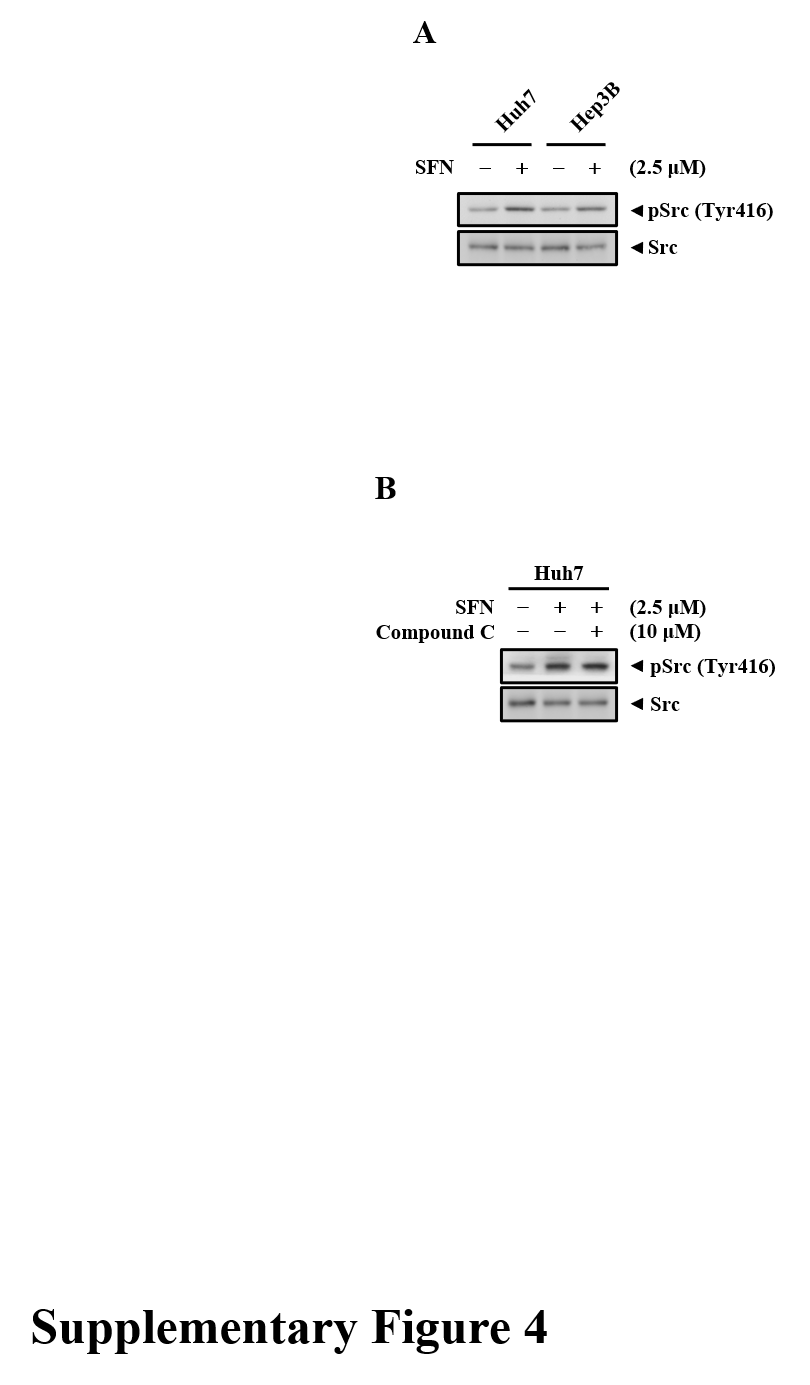

Supplement: Supplementary Figure 4 — The effects of sorafenib and the AMPK inhibitor compound C on Src phosphorylation in liver cancer cells. (A) Two types of liver cancer cells (Huh7 and Hep3B) were treated with or without sorafenib (2.5 μM) for 24 h and harvested for Western blot analyses. (B) Huh7 cells were pretreated with or without the AMPK inhibitor compound C (10 μM) for 0.5 h and then treated with or without sorafenib (2.5 μM) for an additional 6 h. Whole cell lysates were harvested for Western blot analyses and examined indicated proteins with specific antibodies. [file Figure_4.TIF]
