# Supplementary material for: Metformin Resensitizes Sorafenib-Resistant HCC Cells Through AMPK-Dependent Autophagy Activation
Source: Front Cell Dev Biol. 2021 Jan 21;8:596655. doi: 10.3389/fcell.2020.596655 (PMC7931828; doi:10.3389/fcell.2020.596655)
Supplement: Supplementary Table 1 — Animal studies comply with the ARRIVE guidelines. [file Table_1.DOCX]

**Supplementary materials and methods**

***Materials***

TRIzol RNA extraction reagent, SuperScript^TM^ III, Dulbecco's modified Eagle's medium and Opti-MEM medium were obtained from Invitrogen (Carlsbad, CA, USA). Fetal bovine serum was purchased from HyClone Laboratories (Logan, UT, USA). Metformin and chloroquine were purchased from Sigma (St. Louis, MO, USA). Compound C (AMPK inhibitor) was purchased from Calbiochem (San Diego, CA, USA). Antibodies against CEBPD, AMPKα, and p-AMPK (T172) were purchased from Santa Cruz Biotechnology (Santa Cruz, CA, USA). Antibodies against LC3B and p84 were purchased from GeneTex (Irvine, CA, USA). Antibodies against ACC, pACC, ERK 1/2, pERK 1/2, EGFR, pEGFR (Y1068) and Caspase 3 were purchased from Cell Signaling Technology (Beverly, MA, USA).

***Data mining of the liver tissue transcriptome dataset***

From a dataset deposited on GEO (National Center for Biotechnology Information, Bethesda, MD), GSE83148, the raw CEL files from healthy (n = 6) and HBV-infected (n = 122) liver tissues were retrieved and imported into Nexus Expression 3 software to analyze the association of EGFR, CEBPD, and LC3B transcript levels. All hepatitis samples were HBV infected, which was validated by positive HBsAg or serum HBV-DNA. The samples with HCV infection or metabolic liver injury (e.g. fatty liver, chronic alcoholic hepatitis, etc.) were excluded.

***HBx transgenic mice, tissue preparation, and immunofluorescence analysis***

The HBx transgenic mice used in this study were established and described elsewhere [1]. The HBx transgenic mice were bred in a specific pathogen-free environment and all mouse experiments complied with the guidelines in the “Guide for the Care and Use of Laboratory Animals” (NIH publications 86-23 revised 1985) and were approved by the Institutional Animal Care and Use Committee (IACUC) of College of Medicine, National Cheng Kung University (Approval Number: 98129). The HBx transgenic mice developed hepatic tumor after 13 to 16 months of age. The available liver specimens were well-prepared with 5 μm-thick paraffin sections mounted on slides, dried in an oven at 65°C for 15 minutes, and deparaffinized in xylene and ethanol. The sections were then immersed in citrate buffer to perform heat-induced epitope retrieval (HIER). The slides were stained with CEBPD (LifeSpan BioSciences) or LC3B (GeneTex) primary antibody at 4°C overnight. The slides were then incubated with TRITC-conjugated secondary antibody (Jackson ImmunoResearch) at room temperature for 1 hour. The slides were counterstained with 4',6-diamidino-2-phenylindole (DAPI) (Enzo Life Sciences).

***Quantitative real time polymerase chain reaction (Q-PCR)***

Real-time PCR was performed using KAPA SYBR FAST qPCR Master Mix and the CFX95^TM^ Real-Time PCR Detection machine. Primer sequences were as follows: CEBPD: 5’-GCCATGTACGACGACGAGAG-3’ and 5’-TGTGATTGCTGTTGAAGAGGTC-3’; GAPDH: 5’-CCACCCAGAAGACTGTGGAT-3’ and 5’-TTCAGCTCAGGGATGACCTT-3’. Q-PCR was conducted with the following amplification conditions: 1 cycle of 95 °C for 10 min, 39 cycles of 95 °C for 5 sec, 60 °C for 10 sec and 72 °C for 15 sec. Finally, a melting curve was performed to check for the presence of a single product from each reaction. Expression levels of the genes of interest were then compared to expression of GAPDH.

***Methylation-speciﬁc PCR (MSP)***

Genomic DNA was isolated from cell lines using a DNeasy Tissue Kit (QIAGEN). After sodium bisulphite (Zymo research) treatment of genomic DNA, DNA was amplified by PCR using primers specific to methylated sequences. Primer sequences were as follows: the amplification primer pair: 5’-GAGAAGGTTTTGGAGTGTTGGTAGA-3’ and 5’-Biotin-CCCCCTCTCAATTCCTCC-3’; the sequencing primers: 5’-GGTAGAGGGAGTGTTAT-3’ and 5’-GGGAGGGAGTAGTAG-3’. PCR amplification and sequencing primers of the *CEBPD* promoter were designed by PyroMark Assay Design Software 2.0.

***Western blot analysis***

Cell lysates were prepared from control and chemically treated cells. Briefly, cells were lysed in modified RIPA buffer [50 mM Tris-HCl (pH 7.4), 150 mM NaCl, 1 mM EDTA, 1% Nonidet P-40, 0.25% sodium deoxycholate, 1 mM DTT, 1 mM phenylmethylsulfonyl fluoride, 1 µg/ml aprotinin and 1 µg/ml of leupeptin] to be analyzed. Following lysis, the lysates were resolved on an SDS-containing 10% polyacrylamide gel, transferred to polyvinylidene difluoride nylon membrane, and probed with specific antibodies at 4 °C overnight. The specific bands were detected by horseradish peroxidase-conjugated antibody and revealed by an enhanced chemiluminescence (ECL) Western blot system from Pierce (Rockford, IL).

**Supplementary tables**

**Supplementary Table 1. Animal studies comply with the ARRIVE guidelines.**

|  | ITEM |  |
| --- | --- | --- |
| Title | 1 | Xenograft in NOD/SCID mice was conducted to confirm the effect of metformin in sorafenib resensitization. |
| Abstract | 2 | **Background:** Our current results suggest that sorafenib cannot efficiently induce autophagic cell death in Hep3B cells due to the EGFR/ERK-induced reduction in AMPK phosphorylation. The aim of this animal study was to verify whether the combination of sorafenib and metformin elicits a stronger antitumor effect.  **Methods:** Hep3B cells were subcutaneously injected into the right flank of NOD/SCID mice. After 14 days, when macroscopic tumors (50-100 mm^3^) had formed, treatment was given to all groups intraperitoneally every day for four weeks.  **Results:** The combined treatment of sorafenib and metformin significantly enhanced cytotoxicity compared with that induced by sorafenib or metformin treatment alone. Importantly, the LC3B-II/LC3B-I ratio was examined in tumor lysates extracted from these experimental xenografts. The result demonstrated that the LC3B-II/LC3B-I ratio was induced in metformin treatment alone as well as in combination group.  **Conclusions:** Metformin that has the direct effect on the activity of AMPK and autophagy may be a potential combined with sorafenib to overcome sorafenib resistance in HCC. |
| INTRODUCTION | | |
| Background | 3 | Because an ectopic human tumor xenograft mouse model can be used to monitor tumorigenicity and tumor growth easily, many researchers have utilized this model for evaluation of anticancer efficacy. Generally, human cancer cells are subcutaneously injected into the flank or back of NOD/SCID mice. The results can be obtained in a few weeks, whereas the genetically engineered mouse models often require more than a year to develop prior to drug therapy. The effect of a drug on the rate of tumor growth has been reported to often be more predictive of a clinical response than tumor shrinkage/regression [2]. However, a challenge presented with orthotopic models, as compared with subcutaneous models, is the difficulty of following tumor growth [3]. Tumor volumes were estimated using two-dimensional measurements of length and width and were calculated with the formula: [*l* × (*w*)^2^] × 0.52, where *l* is length and *w* is width. Drug-related death and body weight change as parameters of toxicity were determined. Drug-related death was presumed animal deaths within 15 days, and over 20% loss of treated mouse body weight compared to control was considered an adverse effect. |
| Objectives | 4 | To assess the *in vivo* effect of the dual treatment of sorafenib and metformin in a human tumor xenograft mouse model. |
| METHODS | | |
| Ethical statement | 5 | All mouse experiments complied with the guidelines in the “Guide for the Care and Use of Laboratory Animals” (NIH publications 86-23 revised 1985) and were approved by the Institutional Animal Care and Use Committee (IACUC) of College of Medicine, National Cheng Kung University (Approval Number: 103209). |
| Study design | 6 | In this study, n refers to number of animals. The animals were placed randomly into four groups (n=5 per group) as follows: (1) the control group, which received identical volumes of vehicle; (2) the sorafenib treatment group, which was treated with sorafenib at doses of 15 mg/kg/day; (3) the metformin treatment group, which was treated with 250 mg/kg/day metformin; and (4) the combined treatment group, which was injected with sorafenib combined with metformin. |
| Experimental procedures | 7 | 1. Male, six-week-old NOD/SCID mice were obtained from the Laboratory Animal Center of National Cheng Kung University, Tainan, Taiwan. Anesthesia was induced by intraperitoneal injection of ketamine (80 mg/kg) and xylazine (10 mg/kg) mixture. Respiratory rate and effort were monitored and level of anesthesia was assessed by pedal reflex to adjust anesthetic delivery as appropriate to maintain surgical plane. Hep3B cells (5×10^6^) in 0.2 ml PBS were inoculated subcutaneously into the right flank of the mice. After 14 days, when macroscopic tumors (50-100 mm^3^) had formed, treatment was given to all groups intraperitoneally every day for four weeks. The mice were then sacrificed with carbon dioxide at 10% to 30% chamber replacement rate for analyses. Animal weight and tumor dimensions were measured every four days with calipers. 2. All experiments were conducted in the light phase. 3. The animals were bred in laboratory mouse cages. The cage was 27.5 cm long, 15.5 cm wide, and 18.5 cm high. 4. Injectable anesthesia has been preferred in mice, because minimal equipment and training is required and initial costs are lower. Ketamine and xylazine is one of the anesthetic combinations used most frequently in rodents [4]. |
| Experimental animals | 8 | 1. Male NOD/SCID mice (24.72 g), aged 6 weeks, were included (n=20). 2. The NOD.CB17-Prkdc^scid^, also known as NOD/SCID, mice were obtained from the Laboratory Animal Center of National Cheng Kung University, Tainan, Taiwan and acclimatized for at least 48 h. Vendor health reports indicated that the mice were free of known viral, bacterial, and parasitic pathogens. |
| Housing and husbandry | 9 | 1. The animals were maintained in laminar flow cabinets under specific pathogen-free conditions. Prior to surgery the animals were housed with 4-5 companions in laboratory mouse cages filled with Lignocel® (hygienic animal bedding). 2. All food, water and litter were sterilized prior to use. Temperature (20-21°C) and humidity (50-60%) were controlled. Daily light cycles were 12 h light and 12 h dark. All mice were allowed free access to water and diet. 3. The animals were manipulated under sterile conditions. During the postoperative period, incandescent lamp (50-75 watt) was provided for anesthetic recovery. All animals must be continuously monitored until maintaining upright posture and walking normally before return to the animal housing room. |
| Sample size | 10 | Twenty healthy mice were divided into four groups of five each. Sample size calculations were performed in ClinCalc.com before the start of the study that how much minimum difference between two groups can be considered as clinically significant. To achieve power = 0.8 and alpha = 0.05 to detect this difference would require a total of 20 animals. |
| Allocating animals to experimental groups | 11 | 1. The animals were randomly divided into four groups (n=5 per group). 2. To minimize potential confounding factors, the order of treatment and assessment was fixed and the cage location was also consistent. |
| Experimental outcomes | 12 | The primary outcome analyzed was tumor volumes. In addition, the secondary outcome evaluated was autophagy markers. |
| Statistical methods | 13 | 1. The Student's t-test for normally distributed data (e.g., tumor volume and body weight) or the Mann-Whitney U test for non-normally distributed data (e.g., Western blotting) were performed to compare the differences. 2. For each test, the experimental unit was an individual animal. 3. The use of parametric or non-parametric tests was based on results from analyses of distributions (D'Agostino-Pearson omnibus normality test). |
| RESULTS | | |
| Baseline data | 14 | The animals’ health status was monitored throughout the experiments by a health surveillance program according to NIH guidelines. The mice were free of all viral, bacterial, and parasitic pathogens listed in the NIH recommendations. |
| Numbers analyzed | 15 | Because no drug-related death was observed, all animals in each group (5/5) were included in each analysis. |
| Outcomes and estimation | 16 | In accordance with the ARRIVE guidelines, the data were expressed as the means ± SEM. Differences were considered statistically significant when indicated by asterisks. (* = *p* ≤ 0.05, ** = *p* ≤ 0.01, *** = *p* ≤ 0.001) |
| Adverse events | 17 | The mice tolerated the combined treatment well as evidenced by no drug-related death or weight loss was observed after treatment. |
| DISCUSSION | | |
| Interpretation/ scientific implications | 18 | 1. Our current results suggest that sorafenib cannot efficiently induce autophagic cell death in Hep3B cells due to the EGFR/ERK-induced reduction in AMPK phosphorylation. The aim of this animal study was to verify whether the combination of sorafenib and metformin elicits a stronger antitumor effect in a Hep3B cell xenograft mouse model. Consistent with the *in vitro* results, the combined treatment of sorafenib and metformin significantly enhanced cytotoxicity compared with that induced by sorafenib or metformin treatment alone. Importantly, the LC3B-II/LC3B-I ratio was examined in tumor lysates extracted from these experimental xenografts. The result demonstrated that the LC3B-II/LC3B-I ratio was induced in metformin treatment alone as well as in combination group. The RNA binding protein HuR has been suggested to contribute to EGFR abundance by stabilizing EGFR transcripts in Hep3B cells [5]. However, the clinical results revealed that the addition of erlotinib, an oral tyrosine kinase inhibitor of EGFR, to sorafenib did not affect the overall survival [6]. Therefore, metformin that has the direct effect on the activity of AMPK and autophagy may be a potential combined with sorafenib to overcome sorafenib resistance in HCC. 2. Xenografts using human cell lines to test drug responses do not often correlate with clinical activity in patients [7]. 3. Alternatively, human cancer cells could subcutaneously be injected into both flanks of NOD/SCID mice to reduce the number of animals (i.e., n refers to number of xenografts). However, it might be more stressful to the mice. |
| Generalizability/ translation | 19 | Xenograft models of human tumor could be used as a powerful translation research tool for preclinical assessment of anticancer drugs. |
| Funding | 20 | This study was supported by grant MOST106-2320-B-006-063-MY3 from the Ministry of Science and Technology and in part by funding from the Headquarters of University Advancement at National Cheng Kung University, which is sponsored by the Ministry of Education. |

**Supplementary references**

1. Wu, Y.F., et al., *Chemopreventive effect of silymarin on liver pathology in HBV X protein transgenic mice.* Cancer Res, 2008. **68**(6): p. 2033-42.

2. Kelland, L.R., *Of mice and men: values and liabilities of the athymic nude mouse model in anticancer drug development.* Eur J Cancer, 2004. **40**(6): p. 827-36.

3. Jung, J., *Human tumor xenograft models for preclinical assessment of anticancer drug development.* Toxicol Res, 2014. **30**(1): p. 1-5.

4. Arras, M., et al., *Optimization of intraperitoneal injection anesthesia in mice: drugs, dosages, adverse effects, and anesthesia depth.* Comp Med, 2001. **51**(5): p. 443-56.

5. Hung, C.M., et al., *Hepatitis B virus X upregulates HuR protein level to stabilize HER2 expression in hepatocellular carcinoma cells.* Biomed Res Int, 2014. **2014**: p. 827415.

6. Finn, R.S., *Emerging targeted strategies in advanced hepatocellular carcinoma.* Semin Liver Dis, 2013. **33 Suppl 1**: p. S11-9.

7. Kerbel, R.S., *Human tumor xenografts as predictive preclinical models for anticancer drug activity in humans: better than commonly perceived-but they can be improved.* Cancer Biol Ther, 2003. **2**(4 Suppl 1): p. S134-9.
